# Transactivator of Transcription (Tat)-Induced Neuroinflammation as a Key Pathway in Neuronal Dysfunction: A Scoping Review

**DOI:** 10.1007/s12035-024-04173-w

**Published:** 2024-04-17

**Authors:** Tshengedzeni Muvenda, Aurelia A. Williams, Monray Edward Williams

**Affiliations:** Human Metabolomics, North-West University, Potchefstroom, South Africa

**Keywords:** Neurocognitive impairment, Neuroinflammation, Astrocytes, Microglia, Tat, HIV

## Abstract

The activity of HIV-1 and its viral proteins within the central nervous system (CNS) is responsible for a wide array of neuropathological effects, resulting in a spectrum of neurocognitive deficits defined as HIV-associated neurocognitive disorders (HAND). Amongst the various viral proteins, the transactivator of transcription (Tat) remains detectable even with effective antiretroviral therapy (ART) and suppressed viremia, highlighting the significance of this protein in the modern ART era. Tat has been extensively researched in both fundamental and clinical settings due to its role in neuroinflammation, neuronal damage, and neurocognitive impairment amongst people living with HIV (PLHIV). To date, numerous fundamental studies have explored Tat-induced neuroinflammation. However, there is no clear consensus on the most frequently studied inflammatory markers or the consistency in the levels of these Tat-induced inflammatory marker levels across different studies. Therefore, we conducted a scoping review of studies investigating Tat-induced neuroinflammation. We conducted searches in PubMed, Scopus, and Web of Science databases using a search protocol tailored specifically to adhere to the Preferred Reporting Items for Systematic Reviews and Meta-Analyses for scoping reviews (PRISMA-ScR) guidelines. From the 22 included studies, findings suggest that the HIV-1 Tat protein amplifies levels of neuroinflammatory markers. Amongst the vast array of inflammatory markers explored in the included studies, consistent results point to higher levels of CCL2, IL-6, IL-8, and TNF-α in primary cells and cell lines exposed to or transfected with HIV-1 Tat. These markers are regulated by key inflammatory pathways, such as the extracellular signal-regulated kinase (ERK)1/2 mitogen-activated protein kinase (MAPK) pathway, the phosphatidylinositol 3-kinase (PI3K) pathway, the p38 MAPK pathway, and nuclear factor-kB (NF-kB). Furthermore, Tat has been shown to induce neuronal apoptosis, both directly and indirectly. With regards to study designs, utilizing full-length Tat101 at concentrations ranging from 100 to 1000 ng/ml and durations of 24 and 48 h appears optimal for investigating Tat-induced neuroinflammation. In this context, we highlight specific inflammatory markers and pathways that are potentially pivotal in Tat-induced neuroinflammation and subsequent neuronal damage. A deeper investigation into these markers and pathways is crucial to better understand their roles in the development of HAND.

**Supplementary Information:**

The online version contains supplementary material available at 10.1007/s12035-024-04173-w.

## Introduction

Human immunodeficiency virus (HIV) and acquired immunodeficiency syndrome (AIDS) remain major global public health challenges. As of 2021, UNAIDS’ Global HIV & AIDS statistics reported that there were 38.4 million people living with HIV (PLHIV) and 1.5 million diagnosed with AIDS [1]. There are two distinct types of the virus that can lead to AIDS, namely HIV-1 and HIV-2, with HIV-1 being the predominant subtype worldwide [2]. HIV-1 can further be classified into the following four groups: M, O, N, and P. Amongst these, group M is the “major” group, largely responsible for the global human HIV pandemic. Based on genomic variations within group M, nine sub-types are identified by the letters A–D, F–H, J, and K [3, 4]. These HIV-1 subtypes are geographically located in specific regions. HIV-1 subtype B (HIV-1B) is geographically located in America and Europe and accounts for about 12% of all HIV-1 infections [5]. In contrast, the global dominant subtype of HIV-1 is subtype-C (HIV-1C), which is present in countries of Southern Africa and India and represents about 50% of the world’s HIV-infected population [6, 7]. These are followed by subtypes A (10.3%), G (4.6%), and D (2.7%). Subtypes F, H, J, and K combined account for 0.9% of global infections [8].

HIV-1 is well-known for causing systemic infection, but it can also infect the central nervous system (CNS), often leading to neuronal damage [9, 10]. Early in the course of HIV-1 infection, the virus can penetrate the blood–brain barrier (BBB), infecting and/or activating resident brain cells [10] and resulting in a range of neurological impairments termed HIV-associated neurocognitive disorders (HAND) in PLHIV [11]. HAND encompasses a range of neurocognitive impairments, including asymptomatic neurocognitive impairment (ANI), mild neurocognitive disorder (MND), and HIV-1-associated dementia (HAD) [11, 12]. Despite the availability and use of effective combined antiretroviral therapy (cART), it is estimated that approximately 20–50% of PLHIV may still develop milder forms of HAND [12–15].

Although the mechanism(s) leading to HAND are not clearly understood, it is associated with heightened levels of cellular and oxidative stress, disturbances in energy metabolism, glutamate regulation, immune activation, inflammation, and neuronal injury [12]. Neurons are rarely infected with HIV, though neuronal damage is a key feature in the development of HAND [10, 16]. Neurons are vulnerable to direct damage by several viral proteins such as transactivator of transcription (Tat), viral protein R (Vpr), glycoprotein (gp) 120, and negative factor (Nef). Of particular interest is the HIV-1 Tat protein due to its fundamental role in HIV-1 neuropathogenesis. Tat is a small basic protein composed of typically 101 amino acids, weighing approximately 14–16 kDa, and is encoded by two distinct exons [17]. The HIV-1 infected cells, including the CNS cells, actively secrete the Tat protein [18–20]. Tat has also been regarded as a neurotoxin as evidenced by neuronal apoptosis induced by direct exposure of cells to the Tat protein [21–23]. It has been observed that Tat can induce indirect neurotoxicity by triggering the release of neurotoxic substances, including inflammatory cytokines from the glia cells and macrophages [10].

Numerous studies have implicated Tat in the dysregulation of neuroinflammation, causing neuronal damage, and its association with the clinical manifestations of HAND. Despite the plethora of evidence, a clear consensus remains elusive regarding which Tat-induced neuroinflammatory markers and pathways are most frequently impacted. Additionally, it is uncertain whether there is consistency in the direction of the association between Tat presence and the levels of inflammatory markers. Finally, there is no consensus regarding which study design characteristics, such as concentration, duration, and Tat length, may be most suitable for understanding and investigating Tat-induced neuroinflammation in this context.

Therefore, in the current study, we aimed to determine the extent of the available evidence by reviewing the literature on this topic to date as per our specified criteria. This was to determine the value of undertaking a full systematic review and meta-analysis to provide commentary on whether Tat contributes to neuroinflammation as highlighted in fundamental studies. Secondary aims were to (1) explore which specific inflammatory markers are most frequently studied and how they are affected by the presence of Tat, (2) investigate which pathways related to inflammation are commonly examined in the context of Tat’s influence, (3) examine whether variations in Tat amino acids have any impact on neuroinflammation and neuronal health, and (4) which study design characteristics may be most suitable for investigating Tat-induced neuroinflammation.

## Methodology

### Study Design

This narrative scoping review sought to synthesize the existing literature from basic/fundamental studies on Tat-induced neuroinflammation, to provide insights into its potential role in neuronal damage. The diverse study designs present in the current literature prompt questions about the viability of a comprehensive systematic review and meta-analysis. As such, this scoping review serves as a preliminary step to gauge the feasibility and value of a more exhaustive systematic review and meta-analysis in the future. The review was conducted in accordance with the Preferred reporting items for systematic reviews and meta-analyses for scoping reviews (PRISMA-ScR) guidelines.

### Eligibility Criteria

Studies were included if they measured Tat-induced inflammatory markers from brain cells. Therefore, these included investigations of Tat-exposed and/or transfected neuronal cells, brain microvascular endothelial cells (bMVECs), astrocytes, and microglia. We focused on the neuroinflammatory pathways in humans; therefore, only studies that investigated human-derived cells were included (primary cells and/or cell lines). Studies were excluded if they investigated neuroinflammation from any other Tat-exposed/transfected cells that were not CNS-related. Clinical studies were excluded. For comparability, marker measurements needed to be done using enzyme-linked immunosorbent assay (ELISA) (or similar cytokine arrays, e.g., BioPlex, proteome profiler array) and transcript-specific polymerase chain reaction (PCR).

### Data Sources

PubMed, Scopus, and Web of Science databases were searched based on all studies published until 30/10/2023 without publication date limitations. Only studies published in English were included. The full search criteria for each database are included in the supplementary file. The following search terms were applied to PubMed: (Tat [tw] OR Gene Products, tat [mh] tat Gene Products, Human Immunodeficiency Virus [mh]) AND (microglia [mh], or monocytes [mh], or macrophages [mh], OR astrocytes [mh]) AND (HIV associated neurocognitive disorders [mh], OR HAND [tw], OR neurocognitive [tw], OR cogniti* [tw] OR Neuropsychological Tests [mh], OR neuronal damage [tw], OR neuronal apoptosis [tw], OR inflammation [mh], OR Cytokines [mh], OR Chemokines [mh], OR Neurogenic Inflammation [mh], OR neuroinflammation [tw], OR TNF [tw], OR Interleukins [mh], OR interleukins [tw], OR Microglia [mh], OR Monocytes [mh], OR Microglia [mh], OR microglia [tw], OR Monocytes [mh], OR monocyte* [tw], OR sCD163 [tw], OR sCD14 [tw], OR sCD40 [tw], OR Neopterin [mh], OR Interferons [mh]).

**Fig. 1:**
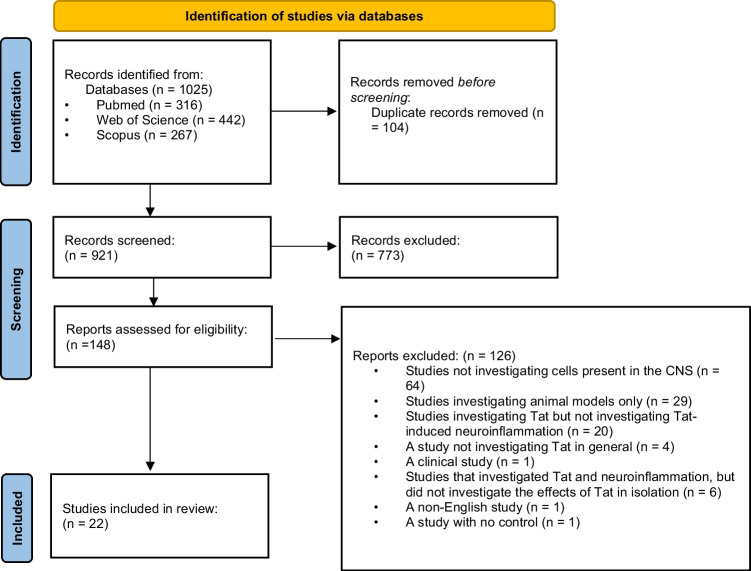
Preferred reporting items for systematic reviews and meta-analyses (PRISMA) flow diagram

Furthermore, reference sections were manually searched, and contact authors of the included studies. The search strategy and the retrieved articles are shown in Fig. 1.

### Data Selection

All articles were retrieved and loaded onto a single database using a reference manager (EndNote X9, Clarivate, PA, USA). Two authors, TM and MEW, independently identified studies meeting the inclusion criteria. Where there was a discrepancy in article inclusion/exclusion, this was discussed amongst the authors, and a decision was made regarding its suitability. Discrepancies were addressed through mutual agreement between the authors. When agreement was not reached, a third author or senior researcher affiliated with the study was consulted for a final decision. Data retrieval covered the type of investigation model, inflammatory markers studied, assays used, and other key findings (Table 1), the various Tat study design parameters investigated across studies (Tat concentration, duration, and length) (Table 1), and Tat-induced pathways and neuronal apoptosis (Table 2). The gathered data were then organized and categorized based on different Tat parameters, inflammatory markers, the assay employed, and the Tat-induced pathways (Tables 1 and 2).

**Table 1:** Summary of studies investigating Tat-induced inflammatory markers, assays employed, and other key findings

Tat status (Tat length)	Model	Markers investigated	Cytokine Assay	Cell viability assay	Dose-response (optimal concentration)	Kinetic response	Key findings for marker levels	Other key findings	Reference
**I. Primary cells**									
Tat treatment (N/A)	Primary human microvascular endothelial cells (MVEC)	Tumour necrosis factor (TNF)-α	Enzyme-linked immunosorbent assay (ELISA)	Terminal deoxynucleotidyl transferase dUTP nick-end labelling (TUNEL) assay	Protein detection, 50 ng/ml; optimal concentration, not determined	Protein detection, 48 h; optimal time, not determined	I. Exposure of MVECs to Tat resulted in elevated TNF-α protein levels compared to untreated controls or those treated with nonviral proteins (e.g., glutathione S-transferase (GST).	I. After cells were exposed to 0.3% concentration of ethanol (EtOH), the levels of TNF-α increased, exceeding those of the untreated control group. II. Additionally, a consistent pattern of reduced TNF-α levels in the presence of Tat treatment was observed when comparing cells treated with 0.3% to 0.1% ethanol concentration; across different HIV strains, R5 (BaL) and X4 (MN) were observed. III. Moreover, whilst Tat augmented the release of ethanol-induced lactate dehydrogenase (LDH), the effect was only modest. Ethanol, on the other hand, did not consistently amplify Tat’s effects.	[24]
Tat treatment (Tat86)	Primary microglia cells	C-C chemokine ligand (CCL)2, IL-8, CXCL10, CCL3, CCL4, and CCL5	ELISA	N/A	Protein detection, 100 ng/ml; optimal concentration, not determined	Protein detection, 24 h; optimal time, not determined	I. Treating microglial cells with 100 ng/ml of Tat increased the secretion of chemokines CCL2, CCL3, CCL4, CCL5, IL-8, and CXCL10 (all *p* < 0.07) compared to untreated cells. II. CCL5 was significantly higher in cells treated with Tat compared to untreated cells; however, its concentration was lower compared to the levels of the other expressed chemokines.	I. In comparison to a Tat mutant, the treatment of microglia with Tat resulted in the secretion of all tested chemokines. II. Tat protein can skew brain chemokine balance, potentially boosting inflammatory cell influx and influencing HIV-1 infection. III. Each chemokine has a distinct regulatory expression. This is particularly clear with CCL5 secretion, as no tested inhibitors curtailed Tat-induced CCL5 production. IV. Moreover, Tat alone can activate microglia.	[25]
Tat treatment (N/A)	Primary microglia cells	TNF-α, IL-1β, IL-6, CCL5, and CCL3	ELISA and PCR	N/A	Protein detection: 0, 10, 30, 100, and 300 ng/ml; optimal concentration, 300 ng/ml; transcript detection 100 ng/ml; optimal concentration, not determined	Protein detection: 8, 24, 48, and 72 h; optimal time: for TNF-α, 8 h; for RANTES, 48 h; for CCL3, 72 h; for IL-6, 24 h; transcript detection, 3 h; optimal time, not determined	I. Primary human foetal microglial cells responded to Tat by producing cytokines (TNF-α, IL-1β, and IL-6) and chemokines (CCL5 and CCL3) in a dose- and time-dependent manner. II. HIV-1 Tat at a concentration of 100 ng/ml also prompted elevated protein levels of TNF-α, IL-1β, IL-6, CCL5, and CCL3 compared to untreated controls. III. Following treatment with Tat at 100 ng/ml, the induced mRNA expression of these cytokines and chemokines was less pronounced.	I. Gp41 more potently stimulated TNF-α, IL-1β, CCL5, and CCL3 production compared to Tat. II. Tat treatment upregulated cytokine (TNF-α, IL-1β, and IL-6) and chemokine (CCL5 and CCL3) mRNA expressions in microglial cells. III. Only Tat treatment, not gp41, induced an increase in intracellular calcium (Ca (2+)) production. IV. Tat treatments did not induce superoxide anion production. VII. HIV-1 replication was not needed for microglial cell activation; Tat alone could trigger cytokine and chemokine production.	[26]
Tat treatment (Tat101)	Primary human microglia	TNF-α, IL-6, and IL-8	ELISA	N/A	Protein detection, 100 nM; optimal concentration, not determined	Protein detection, 8 h; optimal time, not determined	I. Exposure of microglia to Tat led to a significant increase in TNF-α, IL-6, and IL-8 levels compared to untreated cells.	I. Platelet activation in PLHIV could intensify mononuclear phagocyte activation, increasing neurotoxic inflammatory production in the CNS. II. The CD40L-driven rise in monocyte TNF-α production was biologically significant. III. TNF-α was vital for the neurotoxicity of conditioned medium from Tat/CD40L-treated monocytes. IV. CD40 signalling in microglia and monocytes, combined with Tat effects, could escalate central nervous system (CNS) inflammation.	[27]
Tat treatment (Tat72)	Primary astrocytes	CCL2, CCL3, CCL4, and CCL5	ELISA and western blot analysis	N/A	Protein detection: 10, 100, and 1000 nM; optimal concentration, 100 nM	Protein detection, 20 h; optimal time, not determined	I. Exogenous Tat was associated with a dose-dependent increase in the astrocytic release of CCL2; this increase was specific in that the astrocytic release of CCL5, CCL3, and CCL4 was not observed. II. Low nanomolar concentrations of Tat were sufficient to increase astrocytic CCL2 release.	I. Despite 20 h of Tat treatment in doses from 10 to 1000 nM, astrocyte proliferation did not increase. II. Astrocytes stimulated with Tat showed a rise in CCL2-encoding RNA within just 2 h. III. CCL2 release, unrelated to proliferation, could be halted by pre-treating Tat with trypsin or using a specific antibody for immunoadsorption.	[28]
Tat treatment (Tat101)	Primary human astrocytes	CCL2	ELISA	TUNEL Assay	Protein detection, 100 ng/mL; optimal concentration, not determined	Protein detection, 24 h; optimal time, not determined	I. Exposure of astrocytes to Tat significantly increased CCL2 levels compared to untreated controls.	I. Astrocytes significantly influence HIV-induced neurodegeneration. II. Neuronal death was linked to direct or indirect P2X7R activation, with the direct pathway showing greater detrimental effects on neuron survival. III. Tat triggered CCL2 secretion from astrocytes through P2X7R. IV. HIV-1 Tat treatment markedly elevated P2X7R expression in human astrocytes.	[29]
Tat treatment/ transfection (Tat86/Tat101)	Primary human astrocytes	CCL2	ELISA and PCR	N/A	Protein detection, not determined; optimal concentration, not determined	Protein detection, not determined; optimal time, not determined	I. *Lentivirus*-Tat induced a 2.5-fold increase in CCL2 mRNA transcript levels. II. ELISA results showed that *Lentivirus*-Tat approximately doubled the secretion levels of CCL2 from a baseline of 20 ng/ml.	I. In dn-cdk9-transfected cells without Tat stimulation, CCL2 mRNA levels were notably reduced compared to the control, indicating cdk9’s role in the basal expression of these genes. II. In astrocytes expressing Tat, cdk9 binding to the CCL2 promoter was significant. III. Both siRNA and dn-cdk9 inhibited Tat’s induction of CCL2. Yet, treating human astrocytes with an IKK2 inhibitor did not impact Tat’s CCL2 induction.	[30]
**2. Combination of several types of primary cells**									
Tat treatment (Tat72)	Primary neurons/astrocytes	Matrix metalloproteinases (MMP)-1, and urokinase plasminogen activator (uPA)	ELISA	N/A	Protein detection, 100 nM; optimal concentration, not determined	Protein detection, 24 h; optimal time, not determined	In primary neuronal/astrocyte co-cultures, exposure to Tat resulted in a significant increase in MMP-1 and uPA compared to unexposed cells.	I. Tat treatment may boost stimuli production that activates MMP-2 through proteolysis. II. Compared to cells treated with or without pertussis toxin, Tat raised supernatant uPA levels. However, pertussis toxin lessened Tat's impact relative to Tat alone. III. In neuronal/astrocytic cells, Tat also elevated active MMP-2 levels in the supernatant, as seen on gelatine zymograms. IV. Tat prompted the release or activation of MMP-1 and MMP-2 from neural cells.	[31]
Tat treatment (N/A)	Primary BMVEC and astrocytes	TNF-α and IL-8	ELISA	N/A	Protein detection: 10, 50, and 100 ng/ml; optimal concentration. 100 ng/ml	Protein detection, 72 h; optimal time, not determined	I. TNF-α and IL-8 levels were significantly higher in BMVECs exposed to Tat compared to unexposed cells. II. Treatment of cells with 10ng/ml of Tat did not result in a significant increase in TNF levels. III. No statistically significant differences in both TNF-α and IL-8 levels were observed between Tat concentrations at the higher doses of 50 and 100 ng/ml.	I. Exposure to Tat significantly boosted the production of pro-inflammatory cytokines TNF-α and IL-8, potentially worsening the neuropathogenesis process. II. Tat treatment of BMVEC cells led to the release of multiple pro-inflammatory cytokines, capable of inducing p-glycoprotein (P-gp) expression and function.	[32]
Tat treatment (Tat72)	Primary astrocyte and endothelial cells	CCL2, TNF-α, IL-1β, CCL3, CCL4, CCL5, IL-8 and CXCL10	ELISA	N/A	Protein detection, 10 ng/ml	Protein detection, 24 h; optimal time, not determined	I. Astrocytes treated with Tat showed a significant increase in CCL2 levels. II. Using ELISA, the supernatants from Tat-treated cocultures assessed were completely negative for CCL3, IL-8, CCL4, CCL5, and CXCL10 expressions.	I. Tat directly prompted CCL2 expression in astrocytes. II. Tat treatment did not induce TNF-α or IL-1β expression. III. Tat promotes monocyte transmigration through astrocyte-derived CCL2 expression.	[33]
Tat transfection and treatment (Tat72)	Primary human astrocytes and neurons	CCL2	ELISA	MTT (3-[4,5-dimethylthiazol-2-yl]-2,5 diphenyl tetrazolium bromide) assay and TUNEL assay	Protein detection, 100 ng/ml; optimal concentration, not determined	Protein detection, 24 h; optimal time, not determined	I. Exposure of astrocytes to Tat led to a significant increase in CCL2 levels. II. Tat subtype B induced a higher level of CCL2 than Tat subtype C.	I. Whilst the Tat B subtype relied on NF-kB to induce CCL2 production, the Tat C subtype’s induction might be independent of NF-kB. II. Tat C subtype proved less neurotoxic than Tat B, attributed to changes in the dicysteine (C30C31) motif within Tat B’s neurotoxic region. III. Tat-induced neuronal apoptosis was dose-dependent and significantly higher compared to the C-Tat protein, which induced lower levels of neuronal apoptosis.	[34]
**3. Cell lines**									
Tat treatment (Tat86)	Astrocytic cell line (CRT-MG)	CCL2, IL-8, and CXCL10	ELISA and PCR	N/A	Protein detection, 50 nM; optimal concentration, not determined	Protein detection, 24 h; optimal time, not determined	I. Exposing astrocytes to Tat significantly increased both gene transcript and protein levels of CCL2, IL-8, and CXCL10 compared to untreated cells.	I. Tat-stimulated CCL2, IL-8, and CXCL10 protein levels were significantly reduced by Hindsiipropane B, which decreased mRNA levels in CRT-MG cells. II. Hindsiipropane B’s anti-inflammatory effects stem from inhibiting the MAPK/NF-kB/AP-1 signalling axis in Tat-treated cells. III. Hindsiipropane B inhibits HDAC6 expression, a key regulator in Tat-induced chemokine production. IV. Hindsiipropane B reduced HIV-1 Tat-induced reactive oxygen species (ROS) generation and NADPH oxidase activation/expression. V. Hindsiipropane B effectively inhibits HIV-1 Tat-mediated chemokine production via down-regulating the HDAC6-NADPH oxidase-MAPK, NF-kB/AP-1 signalling axis, and could serve as a therapeutic lead compound against HIV-1 Tat-associated neuroinflammation.	[35]
Tat treatment (Tat86)	Astrocytic cell line (CRT-MG)	MMP-9, TNF-α, IL-6, and IL-1β	ELISA and PCR	N/A	Protein detection: 10, 50, 100, 250, 500 and 1000 ng/ml; optimal concentration,1000 ng/ml; transcription detection: 10, 50, 100, 250, 500, and 1000 ng/ml; optimal concentration, 1000 ng/ml	Protein detection: 12, 24, 26, and 48 h; optimal time, 48 h; transcription detection: 1, 3, 6, 9, 12, and 24 h; optimal time, 24 h	I. Treatment of astrocytes with Tat significantly increased both the protein and transcript levels of MMP-9. II. Treatment of astrocytes with Tat led to an increase in the protein levels of TNF-α and IL-6, whilst Tat had no effect on the production levels of IL-1β.	I. Tat significantly boosts MMP-9 expression, which can be notably reduced by neutralizing TNF-α, but not by IL-1β or IL-6. II. Tat upregulates MMP-9 expression through MAPK-NF-kB mechanisms, as well as Tat-induced TNF-α production in astrocytes. III. MMP-9 expression induced by Tat relies on Tat-induced TNF-α production, as pre-incubation with anti-IL-1β or IL-6 antibody had no effect. IV. Pre-incubation with anti-TNF-α antibody decreased Tat-induced levels. MMP-9 expression was observed.	[36]
Tat treatment (Tat72)	Human astrocytoma cell line U373	IL-1β, IL-6, and TNF-α	ELISA and PCR	N/A	Protein detection (primary astrocytes, 100 ng/ml; optimal concentration, not determined; cell line: 10, 100, and 1000 ng/ml; optimal concentration, 1000 ng/ml); transcript detection (cell line: 10, 100, and 1000 ng/ml; optimal concentration, 1000 ng/ml)	Protein detention (primary astrocytes: 5 min, 30 min, and 1 h; optimal time, 5 min; Cell line, 5 min, 30 min, and 1 h; optimal time, 1 h); transcript detection (Cell line, 6 h; optimal time, not determined)	I. IL-6 protein and transcript levels were significantly higher in Tat-treated cultures compared to untreated cells. II. The U373 astrocyte cells did not produce detectable levels of IL-1β protein. III. IL-1β mRNA levels were elevated in U373 cells. IV. Neither IL-1β nor TNF-α protein levels could be determined at any of the time points.	I. Production of IL-1β and IL-6 was not linked to TNF-α production. II. After Tat exposure, astrocytes only produced IL-6, whereas the monocytic cell line (THP-1) induced all three cytokines (IL-1β, IL-6, and TNF-α). III. Tat protein might play a role in increasing IL-1β levels in the brains of HIV dementia patients. Additionally, Tat-treated astrocytes sustained elevated levels of IL-6.	[37]
Tat transfection (Tat24-38)	Human astrocytoma cells (cell line U373 MG)	Interleukin (IL)-1β and CCL2	ELISA	N/A	Protein detection, not determined; optimal concentration, not determined	Protein detection, not determined; optimal time, not determined	I. All Tat clones induced CCL2 and IL-1β compared to non-transfected U373 cells. However, the levels of CCL2 and IL-1β were not significantly different amongst the various Tat clones.	I. Whilst Tat clones triggered IL-1β in THP-1 cells, their induction of CCL2 was limited compared to non-transfected U373 cells. II. Individual Tat clones’ capacity to stimulate host gene expression did not rely on their long terminal repeat (LTR) transactivation potential. III. Tat clones from those with HIV-associated dementia (HAD) had limited HIV-1 LTR transactivation. IV. Clones from individuals without dementia (ND) consistently activated the HIV-1 LTR.	[38]
Tat transfection (N/A)	Astroglia cells (SVGA cell line)	IL-6 and IL-8	Multiplex cytokine assay and PCR	N/A	Protein detection, 200 ng/ml; optimal concentration, not determined	Protein detection, 6, 12, 24, 48, 72, and 96 h; optimal time, not determined	I. In transfected cells, the levels of IL-6 and IL-8 mRNA had increased as early as 1 h, peaked at 6 h, and then steadily declined until 72 h, compared to mock-transfected cells. II. After transfection, IL-6 protein levels began to rise significantly from the 6-h mark and continued to increase, reaching a peak at 96 h, which was the highest observed increase when compared to mock-transfected cells. III. Analogous to IL-6, IL-8 protein expression had also begun its significant rise from 6 h and continued its upward trajectory until 96 h.	I. Tat-mediates induction of both IL-6 and IL-8 in a time-dependent manner in SVG astrocytes.	[39]
Tat transfection (N/A)	Astroglia cells (SVGA cell line)	CCL5	Multiplex Cytokine assay and PCR	N/A	Protein detection, 0.48–0.04 ng/ml; optimal concentration, not determined	Protein detection: 6, 12, 24, 48, and 96 h; optimal time, not determined.	I. Tat induced a time-dependent elevation in CCL5 expression, with peak mRNA and protein levels noted at 1 and 48 h after transfection, respectively. II. CCL5 protein levels exhibited a significant rise as early as 6 h post-transfection. The apex of CCL5 expression was observed at 48 h, followed by a time-dependent decline over the 96-h observation period.	I. Knockdown of both p50 and p65 subunits suggested their involvement in the NF-κB signalling pathway which leads to reduced CCL5 production. II. Pre-treatment with SB203580 did not alter Tat-mediated CCL5 expression at mRNA or protein levels, suggesting p38 MAPK does not participate in NF-κB activation. III. Pre-treatment with inhibitors SC514, LY294002, AG490, and Janex-1 partially decreased Tat-mediated CCL5 induction, hinting at the roles of JAK, PI3K/Akt, and NF-κB in CCL5 expression.	[40]
Tat transfection (N/A)	Astrocytic cell line (HEB)	IL-6 and TNF-α	ELISA and PCR	Annexin V-propidium; iodide (PI) double staining assay.	Protein detection, not determined; optimal concentration, not determined	Protein detection, not determined; optimal time, not determined	I. Tat (subtype B)-significantly increased the expression levels of IL-6 and TNF-α mRNA compared to controls. II. Likewise, ELISA analysis revealed a significant increase in IL-6 and TNF-α protein levels.	I. Silencing NOTCH3 effectively diminished the IL-6 and TNF-α mRNA and the protein expression induced by Tat. II. Applying cell culture media to neuronal cultures led to reduced cell viability.	[23]
Tat treatment (Tat101)	Astrocytic cell line (HEB)	IL-1β, IL-6, TNF-α, CCL2, CCL5, IL-8, and CXCL10	ELISA and PCR	Cell-counting kit-8 (CCK-8) assay	Protein detection, 200 ng/ml; optimal concentration, not determined; transcript detection, 200 ng/ml; optimal concentration, not determined	Protein detection, 12 h; optimal time, not determined; transcript detection (Cell line: 0, 3, 6, 12, and 24 h; optimal time, 6 h	I. Tat dramatically upregulated IL-1β, IL-6, TNF-α, CCL2, CCL5, IL-8, and CXCL10 expression at mRNA levels to varying degrees. II. Similarly, protein levels of IL-1β, IL-6, and TNF-α were significantly higher in Tat-treated cells compared to control-treated cells.	I. Knockdown of ADAM17 significantly abolished Tat-induced IL-1β, IL-6, TNF-α, CCL2, CCL5, and CXCL10 expressions but showed no effect on IL-8 expression	[41]
**4. Primary cells and cell lines**									
Tat treatment (Tat72)	Primary human astrocytes and astroglioma cells (CRT-MG)	CCL2 and IL-8 and CXCL10, CCL5, CCL3, and CCL4	ELISA and PCR	N/A	Protein detection, 50 nm; optimal concentration, not determined; transcription detection (primary cells: 1, 10, and 100 nM; Optimal concentration, 10 nM; cell line: 0.1, 0.5, 2, 10, and 50 nM; optimal concentration, 50 nM)	Protein detection, 24 h; optimal time, not determined; transcription detection: 0, 1, 2, 4, 6, 12, and 24 h; optimal time, 6–12 h	I. Exposure to Tat in primary human adult astrocytes and CRT-MG cells led to a significant increase in both transcript and protein levels of IL-8, CXCL10, and CCL2. II. Tat did not lead to an increase in CCL3 or CCL4 transcript or protein expression in astrocytes. III. Only a minor increase in CCL5 production was observed in cell lines.	I. Tat activation of CCL2 and IL-8 gene expression is only partially dependent on p38 MAPK activation. II. CCL2 and IL-8 mRNAs were constitutively expressed at low levels in primary astrocytes.	[42]
Tat treatment (Tat72)	Primary astrocytes and astrocytoma cell line U373	TNF-α	ELISA	N/A	Protein detection (primary cells: 0, 10, 100, and 1000 nM; optimal concentration, not determined; cell line, 1000 nM; optimal concentration, not determined)	Protein detection (primary cells: 0, 1, 2, 4, 6, 12, and 24 h; optimal time, 1 h; cell line: 0, 1, 2, 4, 6, 12, and 24 h; optimal time, not determined)	I. In primary astrocytes exposed to 1 µM Tat, there was a significant increase in TNF-α levels (*p* < 0.05). The peak levels were attained within 1 h, followed by a subsequent decrease in TNF-α concentrations. In the U373 cell line, TNF-α was undetectable.	I. Tat-induced TNF-α induction was observed to be mediated by NF-kB. II. TNF-α production was lower in astrocytes compared to macrophages after Tat treatment. III. Although Tat could induce TNF-α in astrocytes, the dose required and time profile of TNF-α was different from that in macrophages	[43]
Tat treatment (Tat72)	Primary human astrocytes and cell lines (U-87 and A172)	CXCL10	ELISA and PCR	N/A	Protein detection, not determined; transcript detection, not determined	Protein detection (primary cells, 24 h; optimal time, not determined); transcript detection (Cell line: 3, 6, and 12 h; optimal time, not determined)	I. Tat alone did not induce CXCL10 mRNA transcription or elevate protein levels. The combination of Tat and pro-inflammatory cytokines (IFN-γ and TNF-α) led to the induction of CXCL10 at both the RNA and protein levels in cell lines.	I. Overexpression of CXCL10 can lead to increased inflammatory cell influx into the CNS and exacerbate neuronal dysfunction/death in later-stage HAD. II. Each of the Jnk, p38, and Akt pathways showed mild activation by Tat alone. III. CXCL10 induction was regulated at the transcriptional level by activating the p38, Jnk, and Akt signalling pathways. IV. Cytokines significantly enhanced both CXCL10 RNA and protein induction. V. IFN-γ and TNF-α regulate CXCL10 expression in human astrocytes at both RNA and protein levels. VI. Tat, combined with cytokines (IFN-γ and TNF-α), strongly activates the CXCL10 gene.	[44]

Studies are tabulated according to whether they were treated or transfected with Tat followed by cell types, including (1) primary cells, (2) a combination of several types of primary cells, (3) cell lines, and (4) primary cells and cell lines

**Table 2:** Pathways for Tat-induced neuroinflammation and neuronal apoptosis

Reference	Markers	Pathways for Tat-induced neuroinflammation	Neuronal apoptosis
[24]	Tumour necrosis factor (TNF)-α	N/A	I. Direct: Tat causes apoptosis to BMVECs II. Indirect: N/A
[25]	CCL2, IL-8, CXCL10, CCL3, CCL4, and CCL5	I. CCL2 and CCL4 were mediated by the ERK1/2 MAPK and PI3K pathways. II. IL-8 and CCL3 were influenced solely by the p38 MAPK pathway. III. CXCL10 was partially regulated by the ERK1/2 MAPK, PI3K, and p38 MAPK pathways. IV. CCL5’s expression was not affected by any tested pathway.	N/A
[38]	Interleukin (IL)-1β and C-C chemokine ligand (CCL)-2	N/A	N/A
[21]	CCL2, macrophage inflammatory protein-1α (MIP)-1α, MIP-1β, and CCL5	N/A	N/A
[31]	Matrix metalloproteinases (MMP)-1, and urokinase plasminogen activator (uPA)	N/A	N/A
[35]	CCL2, IL-8, and CXCL10	I. CCL2, IL-8, and CXCL10 were mediated by MAPK, NF-κB, and AP-1.	N/A
[36]	MMP-9, TNF-α, IL-6, and IL-1β	I. MMP-9 was mediated by MAPK-NF-kB mechanisms and Tat-induced TNF-alpha in astrocytes.	N/A
[30]	CCL2	I. CCL2: Induction was independent of the NF-kB classical pathway but was notably inhibited by specific cyclin-dependent kinase 9 (cdk9).	N/A
[42]	CCL2, IL-8 and CXCL10, CCL5, MIP-1α, or MIP-1β	I. CCL2, IL-8, and CXCL10 were mediated by the MAPK pathway	N/A
[32]	TNF-α and IL-8	N/A	N/A
[43]	TNF-α	N/A	N/A
[34]	CCL2	N/A	I. Direct: Tat treatment of primary neuronal cultures with nanogram per millilitre concentration of Tat proteins induced accelerated levels of neuronal death within 24 h in a dose-dependent II. Indirect: Conditioned media from transfected astrocytes induced significant neuronal cell death.
[37]	IL-1β, IL-6, and TNF-α	I. IL-1β, IL-6, and TNF-α were mediated by the NFκB-dependent pathway	N/A
[39]	IL-6 and IL-8	I. IL-6 and IL-8: Both were influenced by NF-kB and AP-1. II. For IL-6, p38δ activated AP-1 III. IL-8: AP-1 was activated by JNK, not p38 MAPK. IV. Both PI3K/Akt and p38 MAPK (β subunit) contributed to NF-κB activation, leading to IL-6 and IL-8 production.	N/A
[40]	CCL5	I. CCL5: NF-κB, AP-1, C/EBPα and C/EBPγ transcription factors, and JAK, PI3K/Akt, and p38 MAPK signalling pathways	N/A
[26]	TNF-α, IL-1β, IL-6, CCL5, and MIP-1α	N/A	N/A
[27]	TNF-α, IL-6, and IL-8	I. TNF-α activated by NF-κB	N/A
[29]	CCL2	I. Tat elevates P2X7R expression, triggering a rise in intracellular calcium, leading to ERK1/2 phosphorylation and subsequent CCL2 release from astrocytes	I. Direct: Neurons directly exposed to Tat showed a significant 36 ± 6% death rate after 24 h compared to control. II. Indirect: Neuronal cell death significantly increased when exposed to Tat-treated astrocyte-conditioned media.
[33]	CCL2, TNF-α and IL-1β, MIP-1α, MIP-1β, CCL5, IL-8, and CXCL10	N/A	N/A
[44]	CXCL10	I. CXCL10 induction was regulated by the activation of p38, Jnk, and Akt pathways, influencing downstream transcription factors NF-κB and STAT-1α	N/A
[23]	IL-6 and TNF-α	I. NOTCH3 signalling facilitated subtype-B Tat-activated NF-κB signalling pathway	I. Indirect: Neuronal cell death significantly increased when exposed to Tat-treated astrocyte-conditioned media at 72 h only.
[41]	IL-1β, IL-6, TNF-α, CCL2, CCL5, and CXCL10	I. A disintegrin and metalloproteinases (ADAMs)17 exacerbates Tat‑induced inflammatory response in a NF‑κB‑dependent manner in HEB astroglia cells	II. Indirect: Neuronal cell death significantly increased when exposed to Tat-treated astrocyte-conditioned media at 72 h only.

Studies that did not investigate pathways for Tat-induced neuroinflammation and neuronal apoptosis were defined as Not Available (N/A)

### Quality Assessment

TM and MEW assessed the quality of the included studies. The quality criterion was adopted from the Joanna Briggs Institute (JBI) critical appraisal tools. Here we have amended the JBI critical appraisal tools by implementing a Likert scale [45] to provide a quantitative measure of study quality. We adopted and amended the quality questions relevant to in vitro and/or in vivo studies. These included four questions related to study design as follows: (1) is it clear in the study what is the “cause” and what is the “effect” (i.e., there is no confusion about which variable comes first)? (2) was there a control group? (3) were there multiple measurements of the outcome both pre and post-intervention/exposure? and (4) were outcomes measured in a reliable way? Each question was rated for 0 = no, 1 = partly, and 2 = yes. All studies with summative ratings between 6 and 8 were classified as high quality. Studies with ratings between 3 and 5 were considered intermediate quality and between 0 and 2 as low quality (Supplementary Table 1). Cohen’s Kappa was employed to assess inter-rater reliability.

## Results

### Study Characteristics

The search strategy yielded a total of 1025 research studies, as indicated in Fig. 1. Duplicates (*n* = 104) were removed, resulting in 921 studies. Thereafter, abstracts and titles were screened and a total of 773 studies were excluded. Of the remaining 148 studies, full-text articles were assessed, and an additional 126 were excluded as described in Fig. 1. Using the specified selection criteria, 22 fundamental studies were eligible for inclusion.

### Quality Assessment of the Included Studies

The kappa was 1.00 which indicates perfect agreement [46] between the two raters as all studies were considered high quality, with clear descriptions of cause and expected outcomes, a control group, the use of multiple investigations (e.g., inflammation and pathway activation), and appropriate measurements and techniques to answer the research question (Supplementary Table 1).

### Study Design of Various Tat Experiments

In the selected studies (*n* = 22), a variety of sample types were analysed. These included primary microvascular endothelial cells (bMVEC), primary astrocytes, several astrocytic cell lines (CRT-MG, HEB, U373 MG, SVGA, U-87, and A172), primary microglia, and primary neurons. The majority of studies investigated astrocytic cell lines *n* = 11; [23, 36, 37, 39–41, 47–51], followed by primary astrocytes *n* = 9 [21, 29, 32, 48–50, 52–54], primary microglia *n* = 3; [25–27], primary bMVEC *n* = 3 [32, 54, 55], primary neurons *n* = 1 [53], and primary neuronal/astrocyte co-cultures *n* = 1 [56] (Table 1).

To quantify cytokine/inflammatory markers, both protein levels and mRNA transcripts were measured. Various methods were employed for this purpose: ELISA (*n* = 12), a combination of ELISA and PCR (*n* = 8), and a combination of multiplex assay and PCR (*n* = 2).

Many studies (73%, *n* = 16/22) utilized Tat treatment on CNS cells. The remaining studies either employed cells transfected with HIV-1 positive Tat constructs (18%, *n* = 4/22) or used a combination of both approaches (9%, *n* = 2/22). Amongst the studies employing Tat treatment on CNS cells, some conducted dose-response and/or kinetics-based experiments to identify the most appropriate Tat treatment conditions for optimal cytokine expression levels. From these studies, only seven studies measured dose responses [21, 32, 36, 37, 48] (Table 1). Different Tat concentrations were used depending on cell type (primary cells vs cell lines), and for measuring protein and transcripts, various assays were employed such as ELISA and PCR.

In both primary cells and cell lines, a concentration range of 100 to 1000 ng/ml or 50–100 nM consistently produced the strongest inflammatory responses at both transcript and protein levels [21, 26, 36, 37, 48, 49] (Table 1). In kinetic-based experiments, both primary cells and cell lines displayed mixed results when measuring transcripts and proteins. For protein detection, optimal incubation times ranged from 8 to 48 h [26, 36]. Meanwhile, for transcript detection, the times varied between 6 and 24 h [36, 48]. Interestingly, some studies reported that primary cells can yield optimal cytokine expression results in as short as 5 min to 1 h [37, 49]. However, the specific response might vary depending on the cytokine [26, 49]. Additionally, optimal inflammatory responses might be influenced significantly by incubation times. It is noteworthy that primary cells show heightened sensitivity compared to cell lines. As such, when using primary cells, lower concentrations and shorter incubation times with Tat might be sufficient to achieve an optimal inflammatory response.

We conducted an evaluation of the Tat length utilized in studies, as it is regarded as a significant factor in Tat’s functioning. Amongst all the studies reviewed, *n* = 6/22 (27%) did not specify the Tat length utilized. The predominant choice was Tat72, with *n* = 8/22 (36%) studies using it, followed by Tat86 utilized in *n* = 3/22 (14%) studies, and Tat101 utilized in *n* = 3/22 (14%) studies. Additionally, one study (*n* = 1/22, 5%) employed a combination of both Tat86 and Tat101 for transfection and treatment studies, whilst another utilized a Tat peptide 24–38 (*n* = 1/22, 5%) (Table 1).

### Tat Induction and Immune Marker Levels

Studies investigated a broad spectrum of markers, including CCL2, CCL3, CCL4, CCL5, CXCL10, IL-1β, IL-6, IL-8, MMP-1, MMP-9, RANTES, TNF-α, and uPA. Amongst these, the most frequently examined markers were CCL2 (*n* = 10/22, 45%), TNF-α (*n* = 10/22, 45%), CCL5 (*n* = 7/22, 32%), IL-6 (*n* = 7/22, 32%), CXCL10 (*n* = 6/22, 27%), IL-8 (*n* = 6/22, 27%), and IL-1β (*n* = 6/22, 27%) (Table 1).

Whilst it is widely established that Tat can directly impact inflammation, there remains uncertainty regarding a consensus on the direction of its association with inflammation levels. Specifically, it is unclear whether Tat presence consistently leads to an increase in the levels of specific inflammatory markers compared to untreated controls across different experiments. To address this, we focused on the most frequently investigated inflammatory markers (chemokines, cytokines, and interleukins) in this field of research to determine if Tat consistently elevates the levels of these inflammatory markers.

**Fig. 2:**
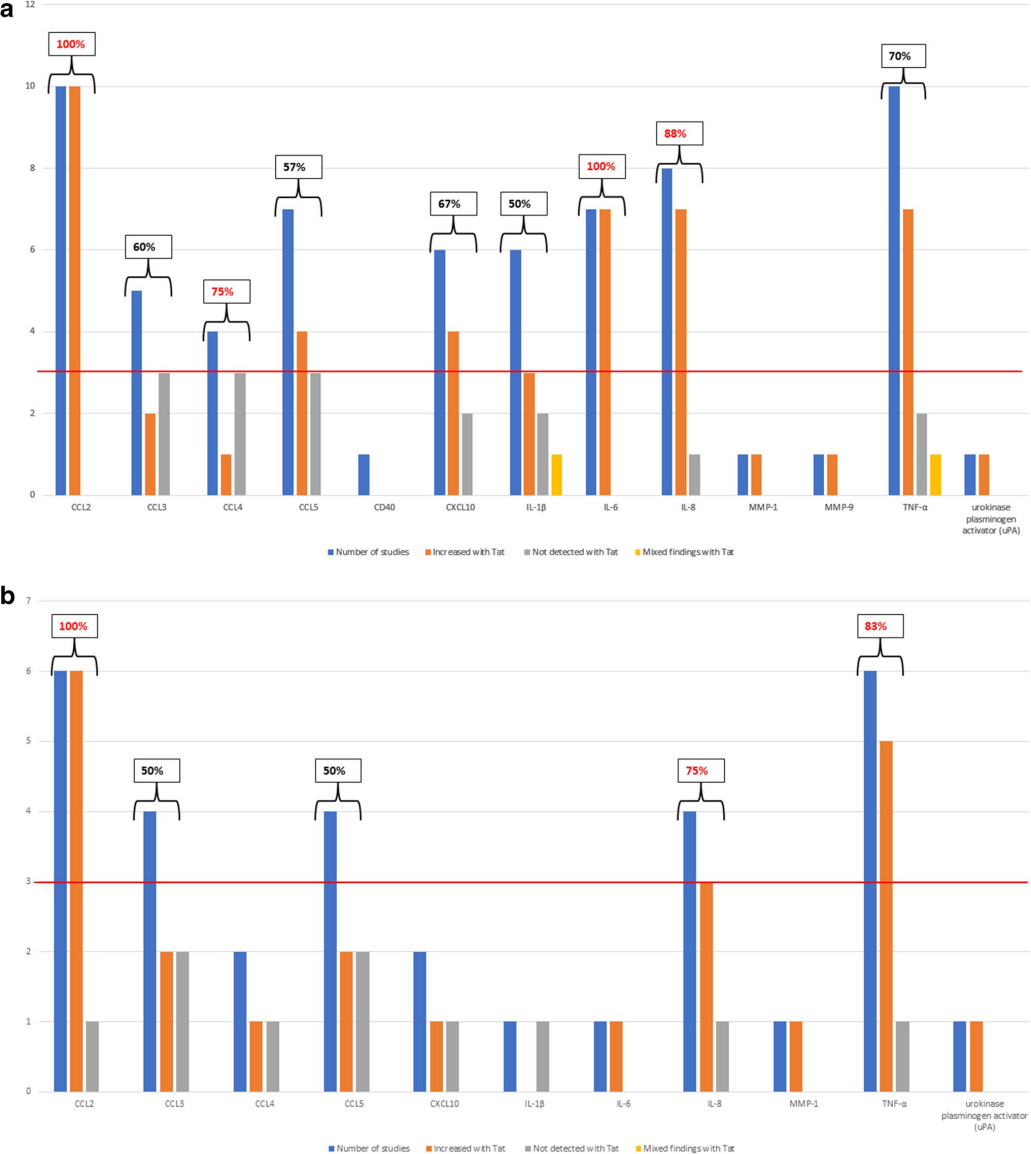
**A** The frequency of inflammatory markers investigated across studies investigating primary cells and cell lines. The inflammatory markers here are ordered as chemokines, interleukins, matrix metalloproteinase, cytokines, and urokinase. **B** The frequency of inflammatory markers investigated across studies investigating primary cells only. The inflammatory markers here are ordered as chemokines, interleukins, matrix metalloproteinase, cytokines, and urokinase

Given that certain markers were studied more frequently, naturally accumulating more supporting evidence, we considered the frequency of investigation when interpreting the findings. In this review, we applied a criterion to identify markers as “noteworthy”, building upon previous approaches [57, 58]. A marker (whether a gene transcript or protein) was considered noteworthy if it met two criteria: (1) it was investigated in three or more independent studies (as shown by the red cut-offline in Fig. 2A) and (2) 75% or more of the studies that examined this marker reported consistent levels when treated or transfected with Tat. If both conditions were met, the marker was deemed a “noteworthy marker” of finding for subsequent research (Fig. 2A). The markers, CCL2, CCL3, CCL4, CCL5, CXCL10, IL-1β, IL-6, IL-8, and TNF-α were investigated by ≥ 3 independent studies, therefore meeting our first criteria (Fig. 2A). CCL2 (10/10, 100%), IL-6 (7/7, 100%), and IL-8 (7/8, 88%) levels in primary cells and/or cell lines were consistently higher in the presence of Tat thus satisfying criteria two. In contrast, whilst reported in ≥ 75% of studies, CCL4 (3/4, 75%) was not detected with Tat presence, thus also satisfying criteria 2 as a noteworthy finding. The levels of CCL3, CCL5, CXCL10, IL-1β, and TNF-α were inconsistent with Tat treatment, thus not meeting our criterion for noteworthy markers. These findings suggest that CCL2, IL-6, and IL-8 may primarily serve as targets of Tat-induced neuroinflammation. For these findings, we did not consider the potential influence of Tat length, as not all studies reported the specific Tat length utilized (*n* = 6, 28% not reported). Furthermore, when attempting to stratify groups according to Tat lengths, there were too few studies available for meaningful comparison.

Considering that inflammatory responses may vary between primary cells and cell lines; we categorized the studies based on primary cells alone to assess whether these markers exhibited consistent levels in response to Tat treatment. Amongst the investigated markers, namely CCL2, CCL3 CCL5, IL-8, and TNF-α were examined by three or more independent studies, meeting our first criterion (Fig. 2B—red cut-offline).

In primary cells, CCL2 (6/6, 100%), IL-8 (3/4, 75%), and TNF-α (5/6, 83%) consistently displayed elevated levels in the presence of Tat, as reported in ≥ 75% of the investigations, meeting the second criteria for noteworthy markers induced by primary cells. Conversely, CCL3 (2/4, 50%) and CCL5 (2/4, 50%) were inconsistent in their detection with Tat presence in primary cells.

Thus, when considering both primary cells and cell lines together, Tat consistently induced higher levels of CCL2, IL-6, and IL-8, as measured by protein and transcript levels. However, when focusing exclusively on primary cells, a consistent elevation was observed for protein/transcripts for CCL2, IL-8, and TNF-α in response to Tat treatment. Across both cell types, higher levels of CCL2 and IL-8 were observed.

### Pathways for Tat-Induced Neuroinflammation

**Fig. 3:**
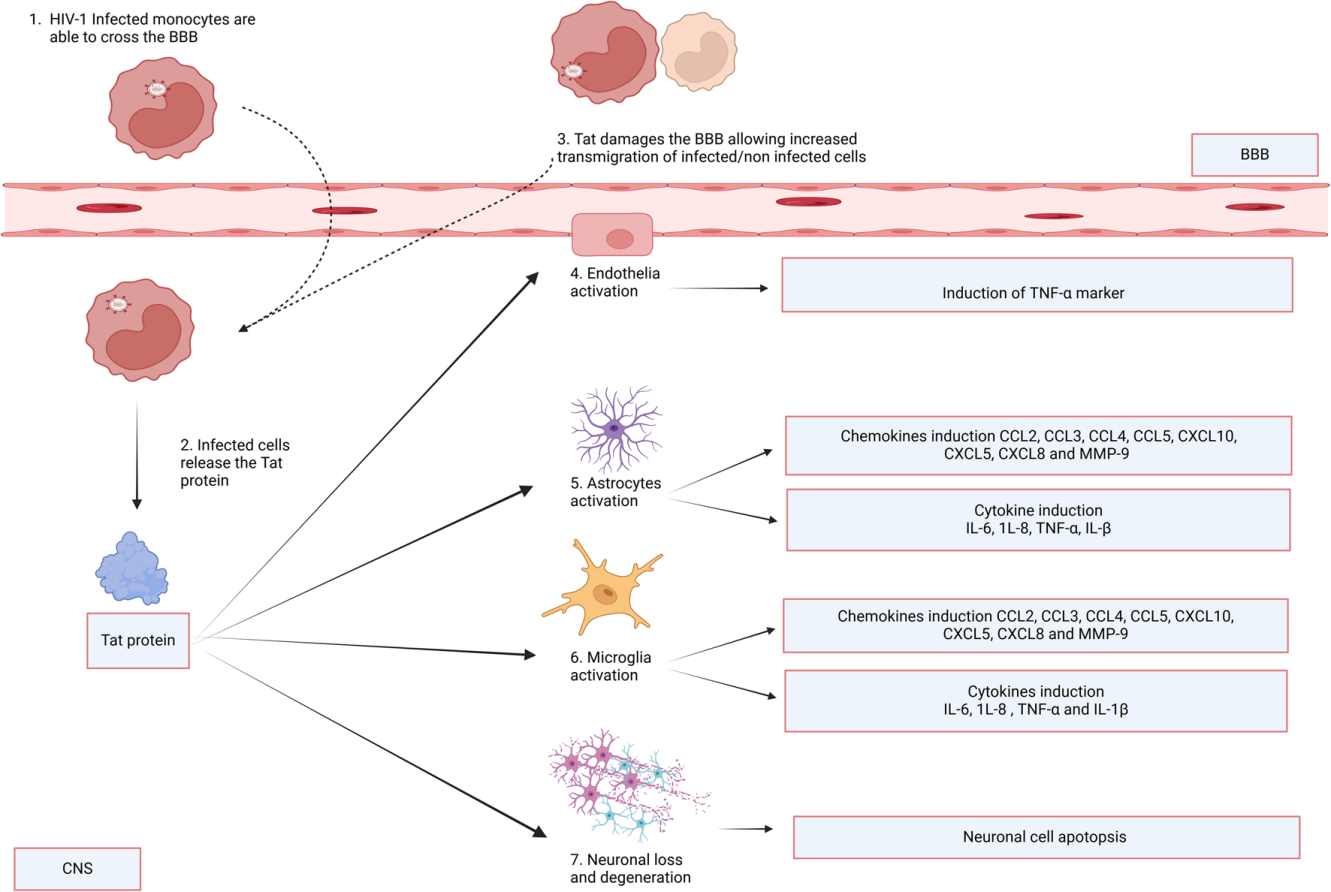
Mechanisms of Tat-induced neuroinflammation. (1) HIV-1 infected monocytes can traverse the blood–brain barrier (BBB). (2) After successfully passing through the BBB, these monocytes initiate the activation of macrophages, which subsequently release viral proteins like the Tat protein. (3) The Tat protein also damages the BBB which allows an increased transmigration of cells into the central nervous system (CNS). This Tat protein, in turn, triggers responses in CNS, including (4) endothelia, (5) astrocytes, and (6) microglia, leading to the induction of neuroinflammation. The activated astrocytes and microglia, through various signalling pathways, induce the production of inflammatory markers. Tat can directly cause damage to (7) neuronal cells

**Fig. 4:**
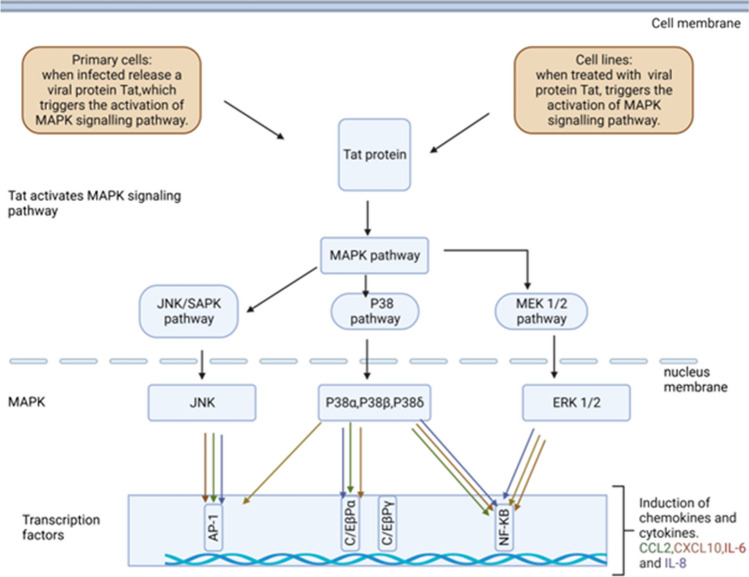
Pathway for Tat induction of inflammatory markers, CCL2, CXCL10, IL-6, and IL-8. Tat induces an inflammatory response in both primary astrocytic cells and various astrocytic cell lines. Exposure to Tat instigates the activation of MAPK kinases (MKKs), which subsequently phosphorylate and activate MAPKs through diverse mechanisms. Amongst the activated MAPKs are extracellular signal-regulated kinase (ERK), c-Jun N-terminal kinase (JNK), and the p38 pathway. Once these MAPKs are activated, transcription factors within the cell nucleus are also triggered. These activated transcription factors, including AP-1 and the NF-κB pathway, bind to specific DNA sequences within the promoter region of astrocytic cells. This binding event leads to the transcription of chemokines and cytokines such as CCL2, CXCL10, IL-6, and IL-8 markers. The heightened transcription of these markers results in increased levels of cytokines and chemokines within the cell

Once HIV crosses the BBB, infected cells release the Tat protein (Fig. 3). The Tat protein can interact with cell various types to induce neuroinflammation (Fig. 3). From all the included studies, *n* = 13/22 studies also investigated the pathways involved in Tat-induced neuroinflammation. These included the pathways for the expression of CCL2 (5/12, 41%), CXCL10 (5/12, 41%), TNF-α (5/12, 41%), IL-6 (5/12, 41%), IL-8 (4/12, 33%), CCL5 (3/12, 25%), IL-1β (3/12, 25%), and at least one study (1/12, 8%) investigating CCL3, CCL4 and MMP9 (Table 2). We also aimed to determine if there was a consensus between studies for the Tat-induced increase of the commonly investigated inflammatory markers. However, fewer studies investigated pathways involved in Tat-induced neuroinflammation. Hence, we therefore looked at markers with findings from at least two independent studies. Collectively, studies reported that CXCL10 was mediated by the extracellular-signal regulated kinase (ERK)1/2 mitogen-activated protein kinase (MAPK) pathway, the phosphatidylinositol 3-kinase (PI3K) pathway, and the p38 MAPK pathway [25, 48, 50, 51]. CCL2 was mediated by the activation of the ERK1/2 MAPK pathway, the PI3K pathway, and nuclear factor-kB (NF-kB) [25, 29, 48, 51, 52]. TNF expression was NF-kappa B-dependent [23, 27, 36, 37, 41]. The findings for CCL5 were mixed, with Nookala and colleagues suggesting that CCL5 was mediated by the JAK, PI3K/Akt, and p38 MAPK signalling pathways and NF-κB, AP-1, C/EBPα, and C/EBPγ transcription factors, whereas Aversa and colleagues suggested that the CCL5 was not mediated by the ERK1/2 MAPK pathway, the PI3K pathway, and nuclear factor-kB (NF-kB). Studies collectively showed the involvement of the p38 MAPK pathway for mediation of IL-8, and IL-6 [25, 27, 36, 37, 39, 48, 51]. For IL-6 and IL-8, expression was mediated by NF-kB and AP-1 transcription factors; however, AP-1 was differentially activated for either cytokine. In the case of IL-6, p38δ activated AP-1, however, JNK was involved in AP-1 activation for IL-8 production. On the other hand, both PI3K/Akt and p38 MAPK (β subunit) were found to be involved in the activation of NF-κB that led to IL-6 and IL-8 production [39] (Fig. 4).

### Tat Sequence Variation, Length, and Neuroinflammation

Most studies have not explored the effect of Tat sequence variation/diversity on neuroinflammation levels. As a result, this review cannot provide commentary on this specific aspect. However, Boven and colleagues used isolates from the brain tissue of participants with HIV-associated dementia (HAD) and non-dementia controls. These clones displayed several sequence variations within the Tat protein. Yet, when comparing HAD with non-HAD, there were no significant differences in CCL2 and IL-1β levels amongst the various Tat clones. The isolates in this study highlighted several amino acid sequence variants. It remains unclear if single-point variations influenced these outcomes since this aspect was not the study’s focus [47].

The study by Mayne and colleagues compared Tat amino acid sequence variations from patients in the Kenyan (subtype A) and Baltimore (subtype B) cohorts. However, they did not study these variants’ effects on neuroinflammation in cell cultures [49]. Mishra and colleagues examined the difference between subtype B and C Tat in inducing inflammation. When astrocytes were exposed to Tat subtypes B and C, the former induced higher CCL2 levels (*p* < 0.003). Moreover, upon transfecting astrocytes with different Tat expression vectors—namely, Tat B (C31) and Tat C (C31S)—a significant induction difference in CCL2 was observed between control cells and Tat B treatment (*p* < 0.0001). In contrast, no such significant difference was observed between vector control cells and Tat C treatment. This suggests that Tat C might have diminished inflammatory effect. Notably, this indicates that the reduction in CCL2 levels with Tat C (CS) might depend on the CS motif’s presence at position 31 [34]. The recent study by Gao and colleagues demonstrated that HEB astroglial cells transfected with pCDNA3.1-flag-Tat-B exhibited significantly higher activity compared to those transfected with pCDNA3.1-flag-Tat-C. These findings suggest that subtype B Tat, rather than subtype C Tat, might interact with the extracellular domain of the NOTCH3 receptor. This interaction potentially triggers the NOTCH3 signalling pathway, which is associated with increased inflammation levels [23]. Collectively, these studies suggested that Tat sequence variation could play a role in influencing neuroinflammation in foundational studies [53]; however, this requires further investigation.

Furthermore, we aimed to evaluate whether the length of Tat utilized in the studies could impact neuroinflammation or the reporting of significant findings. We categorized studies according to Tat72, Tat86, and Tat101. A total of *n* = 8 studies utilized Tat72. From these studies, the findings were variable in that *n* = 5/8 (63%) displayed that at least one of the investigated markers within the respective studies was not detected after treatment/transfection. A total of *n* = 3 studies utilized Tat86. From these studies, the findings were variable in that *n* = 2/3 (66%) displayed that at least one of the investigated markers in the respective studies was not detected after treatment/transfection. This is in contrast to the *n* = 4 studies utilizing Tat101. All studies utilizing Tat 101 and investigated markers in these respective studies were detected after treatment/transfection (Table 1).

### Direct and Indirect Tat-Induced Neuronal Apoptosis

Even though the primary focus of this review was not to determine Tat-induced neuronal apoptosis, we sought to determine if studies investigated the relationship between Tat-induced neuroinflammation and neuronal apoptosis. Only five studies measured direct and indirect cell viability due to Tat presence (Table 2). All studies reported that when Tat was applied to cells of the CNS, there was a significant increase in cell death within 24 h. In addition, using conditioned media exposed to Tat, neuronal apoptosis was also significantly increased, which suggests that Tat-induced neuroinflammation may be a contributor to the observed neuronal apoptosis (Table 2).

## Discussion

In our review, we synthesized findings from 22 fundamental studies exploring Tat-induced neuroinflammation. Several key findings emerge from these studies: (1) Given the heterogeneity in study designs and findings, conducting a comprehensive systematic review analysis at this stage is challenging. However, there is a consensus across studies indicating that soluble Tat is a major contributor to neuroinflammation. (2) Across various studies, elevated levels of CCL2, IL-8, IL-6, and TNF-α have consistently been reported, supported by measurements of both proteins and transcripts. This pattern remains consistent when focusing on studies involving primary cells only after Tat treatment. (3) Tat-induced neuroinflammation is associated with specific pathways, including the ERK1/2 MAPK pathway, the PI3K pathway, the p38 MAPK pathway, and NF-kB. (4) It is important to note that variations in the Tat amino acid sequence may influence the degree of inflammation and subsequent neuronal consequences. (4) Finally, utilizing full-length Tat101 at concentrations ranging from 100 to 1000 ng/ml and durations of 24 and 48 h appears optimal for investigating Tat-induced neuroinflammation.

Firstly, from the studies reviewed, it is evident that Tat plays a significant role in neuroinflammation, which is a primary contributor to the emergence of HAND in the modern ART era. The findings from this research indicate that in primary cells, Tat was linked with elevated levels of markers such as CCL2, IL-6, IL-8, and TNF-α post-Tat treatment. Notably, CCL2 is recognized for its role in neuroinflammation. Consistent with our results, elevated levels of CCL2 have been reported both in the periphery [59–61] and cerebrospinal fluid (CSF) levels in PLHIV with HAND [62, 63]. Additionally, Tat-induced CCL2 plays a crucial role in the transmigration of HIV-infected cells across the BBB, which is a significant neuropathogenic mechanism of HAND [64]. Nonetheless, some argue that CCL2 is vital for the initiation but not the persistence of HIV-mediated neurocognitive disease. This viewpoint aligns with previous research suggesting that CCL2 plays a more foundational role in initiating neuropathophysiological impacts in the initial stages rather than in the later stages of infection [65]. In recent systematic reviews conducted by our group, imbalances in levels of IL-6, IL-8, and IL-10 in the periphery [66] and CNS [67] were the only markers consistently linked with HAND. However, our findings did not take into account the specific Tat length utilized in each study to identify the key neuroinflammatory markers, primarily due to the limited number of studies available for analysis. Therefore, it is essential to interpret these findings with the recognition that Tat length may have influenced the reported results in each study and, consequently, the conclusions drawn from this review.

The HIV-1 Tat has a strong capability to stimulate the production of TNF-α in cells [68]. HIV replication is enhanced by the abundance of TNF-α receptors which is increased in the brain cells of PLHIV [69]. However, primary cells induce high levels of TNF-α after Tat treatment. Mayne and colleagues demonstrated that primary cells indeed induce TNF-α in response to Tat treatment, though at significantly lower levels compared to the other cell types they investigated [70]. Interestingly, in prior research, the correlation between TNF-α and clinical HAND has been inconsistent [66, 67]. This inconsistency might be attributed to TNF-α being a pivotal immunomodulator, potentially operating secondarily in inflammation pathways more linked to HAND. However, here, we provide additional evidence that TNF-α was consistently associated with Tat presence in CNS cells.

Further, we also found that primary cells were more sensitive to Tat exposure compared to cell lines included in this review. The heightened sensitivity observed in primary cells, relative to cell lines, can be attributed to multiple factors that impact the behaviour of cultured microglia. Primary microglia cultures might not represent pure microglial populations; the presence of lingering astrocytes or oligodendrocytes could modify the primary microglia’s susceptibility to insults and inflammatory triggers [71]. Additionally, cytokine responses and inflammation-induced subnetworks might vary depending on the species from which the primary microglia cells are derived [72]. In contrast, immortalized cell lines exhibit an inflammatory response that differs from that of primary cultures. Moreover, these cell lines are prone to genetic drift and morphological changes [71], factors that can further influence their patterns of activation and inflammation.

Primary cells are directly sourced from living tissues, such as skin, blood, or organs. They closely resemble the physiological properties of cells in their natural, in vivo state, including similar morphology, markers, and functions [73]. When these primary cells are subjected to transfection, they can also receive recombinant DNA encoding sensors for measuring cell signalling and promoting cell survival. On the other hand, cell lines typically originate from primary cells that have undergone transformation to allow them to proliferate indefinitely. During this transformation, they often experience genetic and epigenetic changes, which make them less representative of in vivo cells. Furthermore, immortalized cell lines tend to accumulate genetic abnormalities with each passage, thus limiting their utility and affecting the overall reliability of experiments [74]. This also renders them less responsive to cellular stress and insults. In contrast, primary cells have not been subjected to extended culture, retaining a genomic profile that closely mirrors in vivo cells. Over time, cell lines may accumulate genetic mutations that enable them to thrive in laboratory conditions. Nonetheless, they retain many of the metabolic and physiological characteristics of the tissue from which they originate, making them more sensitive and responsive, resulting in heightened sensitivity in experiments.

Secondly, several critical inflammatory pathways have been pinpointed as mediators of Tat-induced neuroinflammation. These include the ERK1/2 MAPK pathway, the PI3K pathway, the p38 MAPK pathway, and the NF-kB pathway. HIV-1 Tat initiates activation of MAPK signalling cascades in which ERK, JNK, and p38 are phosphorylated, and they participate in the production of pro-inflammatory mediators [36, 42]. Tat has been demonstrated to be capable of activating the MAPK signalling pathway in glial cells [75]. Kutsch and colleagues demonstrated that Tat activation of CCL2 and IL-8 gene expression is only partially dependent on p38 MAPK activation. However, Tat-mediated IL-8 gene regulation is stringently controlled by the ERK1/2 pathway, whilst IP-10 regulation by Tat (72 amino acid variant) exclusively involves the p38 MAPK pathway. Additionally, Tat-induction of cytokine production is likely NFκB-dependent. The NF-κB is a major transcription factor which is involved in regulating the expression of many cytokines and chemokines. Whilst the precise molecular mechanism by which HIV-1 Tat-induces NF-κB activation remains unclear, it is possible that the association of target molecules at cell surfaces with extracellular HIV-1 Tat protein may initiate signalling pathways that result in NF-κB activation. It is of particular interest that all three MAPKs engage in the activation of NF-κB in HIV-1 Tat-stimulated human astrocytes [76, 77]. MAPKs are important upstream signalling molecules that can result in the activation of many cytokines mediated through NF-κB activation. Furthermore, Tat activation of CCL2 and IL-8 gene expression was found to be only partially dependent on p38 MAPK activation. Moreover, further investigation is needed to determine the involvement of NF-κB in the induction of the monitored chemokines by HIV-1 Tat in astrocytes and possible MAPK signalling pathways. Therefore, it is crucial to conduct further investigations into MAPK signalling pathways that activate NF-κB activation in primary cells and cell lines because Tat may not be acting through a single receptor-mediated mechanism, and its means of entry may contribute to the effects of Tat.

Thirdly, preliminary findings hint that Tat amino acid sequence variations might influence the extent and nature of neuroinflammation, the prevalence of HAND, and potentially the underlying pathway [78]. Mishra and colleagues investigated the clade-specific differences in neurotoxicity of HIV-1 between clade B and C on human neurons. Their findings strongly suggest that there exists a varying extent of neuronal damage caused by Tat B and Tat C [78]. This variability can be ascribed to the variation of a single amino acid difference at position 31 within the neurotoxic region of the Tat protein. However, the pathogenic significance of the dicysteine motif in Tat could potentially sustain the differential neuronal damage observed in Tat variants which originates from clades B and C. The natural variation in the dicysteine motif of Tat C could potentially be a key factor contributing to these differences. Furthermore, studies not included here demonstrated that Tat amino acid sequence variation influences inflammation in cell culture (macrophages) [79] and clinical samples [80–82]. However, the investigation of Tat sequence variation and inflammation from cells in CNS remains limited and requires further investigation.

We demonstrate that Tat not only directly causes neuronal apoptosis but also induces neuronal apoptosis through dysregulation of inflammation. Given that inflammation is a fundamental pathway in the development of HAND, it is crucial to understand how these networks are regulated. It is also important to note that there is a pressing need for a more in-depth exploration of the highlighted inflammatory markers and pathways. Such efforts would offer a clearer understanding of their functional roles in the onset and progression of HAND.

Finally, for investigations into inflammatory responses, Tat concentrations ranging from 100 to 1000 ng/ml and time points 24 and 48 h appear optimal. Importantly, primary cells have displayed a heightened sensitivity in comparison to cell lines. Therefore, when employing primary cells, researchers might consider using lower Tat concentrations and shorter incubation periods to achieve the desired inflammatory response. Regarding Tat length and studies employing truncated versions of Tat (Tat72 and Tat86), these investigations noted that certain markers were not detected following treatment with these truncated forms. In contrast, studies utilizing Tat101 consistently reported the detection of all investigated markers after Tat101 treatment. Therefore, it remains uncertain whether the truncated versions of Tat were insufficient to activate the inflammatory pathways associated with the investigated markers, or if Tat, in general, did not affect the activation of those pathways. However, in a study that treated an astrocytic cell line, neither IL-1β nor TNF-α protein levels could be determined at any of the time points after treatment with Tat72 [37]. Conversely, in another study that treated an astrocytic cell line, IL-1β and TNF-α were significantly increased after Tat101 treatment [41]. It is therefore reasonable to speculate that full-length Tat101 might provide the most suitable framework for comprehensively understanding the neuroinflammatory profile induced by Tat. Additionally, prior research has suggested that full-length Tat (Tat101) is more commonly detected in individuals living with HIV and may provide the most biological relevance [83]. Therefore, investigations utilizing Tat101 in cell culture may yield findings with greater biological relevance to clinical models [83].

## Recommendations

Based on the findings presented in this review, we can offer several recommendations. Firstly, amongst the numerous studies included, there was a notable variance in study design, which impacted our ability to extract data seamlessly. Moreover, from the consensus drawn in this review, it is evident that future studies of a similar nature could utilize these findings to guide subsequent research.

There is a limited body of work examining the influence of Tat sequence variation on neuroinflammation within CNS cells. Most findings are based on prior studies involving macrophages or non-CNS cells. There is a significant gap in understanding the effects of subtype C Tat on these mechanisms. This is noteworthy since most studies have been conducted in geographical regions dominated by subtype B (e.g., the USA).

Lastly, we suggest that future research includes mediation analyses to discern the relationships between Tat presence, neuroinflammation, and neuronal damage. There is an underlying query as to whether neuroinflammation directly correlates with the extent of neuronal damage. Currently, this relationship is often examined in isolation and deserves a more integrated approach.

## Conclusion

From the available evidence, we highlight the significant role of the HIV-1 viral protein Tat in neuroinflammation. Numerous studies have indicated elevated levels of CCL2, IL-6, TNF-α, and IL-8, as supported by protein and transcript measurements. When exclusively examining studies with primary cells, CCL2, IL-8, and TNF-α levels consistently exhibit an increase after Tat treatment. The induction of neuroinflammation by Tat involves specific pathways, including the extracellular signal-regulated kinase (ERK)1/2 mitogen-activated protein kinase (MAPK) pathway, the phosphatidylinositol 3-kinase (PI3K) pathway, the p38 MAPK pathway, and NF-kB. Additionally, variations in the Tat sequence may affect inflammation and the resulting neuronal consequences. With regards to study designs, utilizing full-length Tat101 at concentrations ranging from 100 to 1000 ng/ml and durations of 24 and 48 h appears optimal for investigating Tat-induced neuroinflammation. We believe that this study provides a comprehensive yet concise review of a widely investigated area, offering researchers key insights into the most commonly studied markers and those most frequently influenced by Tat. Finally, we believe that this review equips researchers with a panel of markers that could serve as the foundation for future research, alleviating the need for exhaustive literature searches in the field. The markers and pathways driven by Tat, as discussed in this review, warrant further exploration to ascertain their potential contributions to the neuropathogenesis of HAND.

## Supplementary Information

Below is the link to the electronic supplementary material.

Supplementary file1 (DOCX 13 KB)

Supplementary file2 (DOCX 43 KB)

## Data Availability

All data is attached to this article.

## Abbreviations

AIDS: Acquired immunodeficiency syndrome
ART: Antiretroviral therapy
ANI: Asymptomatic neurocognitive impairment
BMVEC: Brain microvascular endothelial cells
CCL: C-C chemokine ligand
CCK-8: Cell-counting kit-8
CNS: Central nervous system
Cart: Combined antiretroviral therapy
ELISA: Enzyme-linked immunosorbent assay
(ERK)1/2: Extracellular signal-regulated kinase 1/2
GST: Glutathione S-transferase
(Gp) 120: Glycoprotein 120
HAD: HIV-1-associated dementia
HIV-1B: HIV-1 subtype B
HIV-1C: HIV-1 subtype-C
HAND: HIV-associated neurocognitive disorders
HIV: Human immunodeficiency virus
IL: Interleukin
LTR: Long terminal repeat
MMP-1: Matrix metalloproteinases 1
MVEC: Microvascular endothelial cells
MND: Mild neurocognitive disorder
MAPK: Mitogen-activated protein kinase
Nef: Negative factor
NF-Kb: Nuclear factor-Kb
PLHIV: People living with HIV
PI3K: Phosphatidylinositol 3-kinase
PCR: Polymerase chain reaction
PRISMA-Scr: Preferred reporting items for systematic reviews and meta-analyses for scoping reviews
PI: Propidium iodide
TUNEL: Terminal deoxynucleotidyl transferase Dutp nick-end labelling
Tat: Transactivator of transcription
TNF-A: Tumour necrosis factor A
Vpr: Viral protein R
BBB: Blood–brain barrier
CSF: Cerebrospinal fluid
DNA: Deoxyribonucleic acid
MMP-9: Matrix metalloproteinases 9
JNK: Jun N-terminal kinase
uPA: Urokinase-type plasminogen activator

## References

[1] UNAIDS (2021) UNAIDS DATA 2021. Geneva: Joint United Nations Programme on HIV/AIDS 12349391

[2] Nastri BM, Pagliano P, Zannella C, Folliero V, Masullo A, Rinaldi L, Galdiero M, Franci G (2023) HIV and drug-resistant subtypes. Microorganisms 11(1):221 36677513 10.3390/microorganisms11010221 PMC9861097

[3] de Arellano ER, Alcamí J, López M, Soriano V, Holguín Á (2010) Drastic decrease of transcription activity due to hypermutated long terminal repeat (LTR) region in different HIV-1 subtypes and recombinants. Antivir Res 88(2):152–159 20713090 10.1016/j.antiviral.2010.08.007

[4] Hemelaar J (2012) The origin and diversity of the HIV-1 pandemic. Trends Mol Med 18(3):182–192. 10.1016/j.molmed.2011.12.001 22240486 10.1016/j.molmed.2011.12.001

[5] Taylor BS, Sobieszczyk ME, McCutchan FE, Hammer SM (2008) The challenge of HIV-1 subtype diversity. N Engl J Med 358(15):1590–1602 18403767 10.1056/NEJMra0706737 PMC2614444

[6] Gartner MJ, Roche M, Churchill MJ, Gorry PR, Flynn JK (2020) Understanding the mechanisms driving the spread of subtype C HIV-1. EBioMedicine 53:102682. 10.1016/j.ebiom.2020.102682 10.1016/j.ebiom.2020.102682 PMC7047180 32114391

[7] Bbosa N, Kaleebu P, Ssemwanga D (2019) HIV subtype diversity worldwide. Curr Opin HIV AIDS 14(3):153–160. 10.1097/coh.0000000000000534 30882484 10.1097/COH.0000000000000534

[8] Hemelaar J, Elangovan R, Yun J, Dickson-Tetteh L, Fleminger I, Kirtley S, Williams B, Gouws-Williams E, Ghys PD (2019) Global and regional molecular epidemiology of HIV-1, 1990–2015: a systematic review, global survey, and trend analysis. Lancet Infect Dis 19(2):143–155. 10.1016/s1473-3099(18)30647-9 30509777 10.1016/S1473-3099(18)30647-9

[9] Ojeda-Juárez D, Harahap-Carrillo IS, Kaul M (2023) Neurodegeneration Associated with HIV-1 in the Era of cART. In: Kostrzewa RM (ed) Handbook of Neurotoxicity. Springer International Publishing, Cham, pp 1999-2028. 10.1007/978-3-031-15080-7_137

[10] González-Scarano F, Martín-García J (2005) The neuropathogenesis of AIDS. Nat Rev Immunol 5(1):69–81. 10.1038/nri1527 15630430 10.1038/nri1527

[11] Antinori A, Arendt G, Becker JT, Brew BJ, Byrd DA, Cherner M, Clifford DB, Cinque P, Epstein LG, Goodkin K, Gisslen M, Grant I, Heaton RK, Joseph J, Marder K, Marra CM, McArthur JC, Nunn M, Price RW, Pulliam L, Robertson KR, Sacktor N, Valcour V, Wojna VE (2007) Updated research nosology for HIV-associated neurocognitive disorders. Neurology 69(18):1789–1799. 10.1212/01.WNL.0000287431.88658.8b 17914061 10.1212/01.WNL.0000287431.88658.8b PMC4472366

[12] Saylor D, Dickens AM, Sacktor N, Haughey N, Slusher B, Pletnikov M, Mankowski JL, Brown A, Volsky DJ, McArthur JC (2016) HIV-associated neurocognitive disorder - pathogenesis and prospects for treatment. Nat Rev Neurol 12(5):309. 10.1038/nrneurol.2016.53 27080521 10.1038/nrneurol.2016.53 PMC5842923

[13] Heaton RK, Franklin DR, Ellis RJ, McCutchan JA, Letendre SL, LeBlanc S, Corkran SH, Duarte NA, Clifford DB, Woods SP (2011) HIV-associated neurocognitive disorders before and during the era of combination antiretroviral therapy: differences in rates, nature, and predictors. J Neurovirol 17:3–16 21174240 10.1007/s13365-010-0006-1 PMC3032197

[14] Nightingale S, Winston A, Letendre S, Michael BD, McArthur JC, Khoo S, Solomon T (2014) Controversies in HIV-associated neurocognitive disorders. Lancet Neurol 13(11):1139–1151 25316020 10.1016/S1474-4422(14)70137-1 PMC4313542

[15] Wang Y, Liu M, Lu Q, Farrell M, Lappin JM, Shi J, Lu L, Bao Y (2020) Global prevalence and burden of HIV-associated neurocognitive disorder: a meta-analysis. Neurology 95(19):e2610–e2621. 10.1212/wnl.0000000000010752 32887786 10.1212/WNL.0000000000010752

[16] Agbey C, Avdoshina V, Mocchetti I (2023) Neuronal Cytoskeleton and HIV-Mediated Neurodegeneration. In: Kostrzewa RM (ed) Handbook of Neurotoxicity. Springer International Publishing, Cham, pp 2043-2058. 10.1007/978-3-031-15080-7_230

[17] Williams ME, Zulu SS, Stein DJ, Joska JA, Naudé PJ (2020) Signatures of HIV-1 subtype B and C Tat proteins and their effects in the neuropathogenesis of HIV-associated neurocognitive impairments. Neurobiol Dis 136:104701 31837421 10.1016/j.nbd.2019.104701

[18] Gurwitz KT, Burman RJ, Murugan BD, Garnett S, Ganief T, Soares NC, Raimondo JV, Blackburn JM (2017) Time-dependent, HIV-Tat-induced perturbation of human neurons in vitro: towards a model for the molecular pathology of HIV-associated neurocognitive disorders. Front Mol Neurosci 10:163 28611588 10.3389/fnmol.2017.00163 PMC5447036

[19] Nath A (2002) Human immunodeficiency virus (HIV) proteins in neuropathogenesis of HIV dementia. J Infect Dis 186(Supplement_2): S193-S198 10.1086/344528 12424697

[20] Mele AR, Marino J, Chen K, Pirrone V, Janetopoulos C, Wigdahl B, Klase Z, Nonnemacher MR (2018) Defining the molecular mechanisms of HIV-1 Tat secretion: PtdIns(4,5)P(2) at the epicenter. Traffic. 10.1111/tra.12578 29708629 10.1111/tra.12578 PMC6207469

[21] Conant K, Garzino-Demo A, Nath A, McArthur JC, Halliday W, Power C, Gallo RC, Major EO (1998) Induction of monocyte chemoattractant protein-1 in HIV-1 Tat-stimulated astrocytes and elevation in AIDS dementia. Proc Natl Acad Sci USA 95(6):3117–3121. 10.1073/pnas.95.6.3117 9501225 10.1073/pnas.95.6.3117 PMC19704

[22] Khan IA, Worrad AH, Singh MV, Maggirwar SB, Singh VB (2022) Human immunodeficiency virus-1 Tat exerts its neurotoxic effects by downregulating Sonic hedgehog signaling. J NeuroVirol 28(2):305–311. 10.1007/s13365-022-01061-8 35181862 10.1007/s13365-022-01061-8 PMC9187557

[23] Gao L, Sun W, Zhang D, Shang Y, Li L, Tao W, Zhang L, Liu H (2023) HIV-1 subtype B Tat enhances NOTCH3 signaling in astrocytes to mediate oxidative stress, inflammatory response, and neuronal apoptosis. J Neurovirol 29(4):479–491. 10.1007/s13365-023-01151-1 37358698 10.1007/s13365-023-01151-1

[24] Acheampong E, Mukhtar M, Parveen Z, Ngoubilly N, Ahmad N, Patel C, Pomerantz RJ (2002) Ethanol strongly potentiates apoptosis induced by HIV-1 proteins in primary human brain microvascular endothelial cells. Virology 304(2):222–234 12504564 10.1006/viro.2002.1666

[25] Aversa TGD, Yu KO, Berman JW (2004) Expression of chemokines by human fetal microglia after treatment with the human immunodeficiency virus type 1 protein Tat. J Neurovirol 10(2):86–97 15204927 10.1080/13550280490279807

[26] Sheng W, Hu S, Hegg C, Thayer SA, Peterson P (2000) Activation of human microglial cells by HIV-1 gp41 and Tat proteins. Clin Immunol 96(3):243–251 10964543 10.1006/clim.2000.4905

[27] Sui Z, Sniderhan LF, Schifitto G, Phipps RP, Gelbard HA, Dewhurst S, Maggirwar SB (2007) Functional synergy between CD40 ligand and HIV-1 Tat contributes to inflammation: implications in HIV type 1 dementia. J Immunol 178(5):3226–3236 17312171 10.4049/jimmunol.178.5.3226

[28] Conant K, Garzino-Demo A, Nath A, McArthur JC, Halliday W, Power C, Gallo RC, Major EO (1998) Induction of monocyte chemoattractant protein-1 in HIV-1 Tat-stimulated astrocytes and elevation in AIDS dementia. Proc Natl Acad Sci USA 95 (6):3117–3121. 10.1073/pnas.95.6.3117 10.1073/pnas.95.6.3117 PMC19704 9501225

[29] Tewari M, Monika N, Varghese RK, Menon M, Seth P (2015) Astrocytes mediate HIV-1 Tat-induced neuronal damage via ligand-gated ion channel P2X7R. J Neurochem 132(4):464–476 25272052 10.1111/jnc.12953

[30] Khiati A, Chaloin O, Muller S, Tardieu M, Horellou P (2010) Induction of monocyte chemoattractant protein-1 (MCP-1/CCL2) gene expression by human immunodeficiency virus-1 Tat in human astrocytes is CDK9 dependent. J Neurovirol 16(2):150–167 20370601 10.3109/13550281003735691

[31] Conant K, Hillaire CS, Anderson C, Galey D, Wang J, Nath A (2004) Human immunodeficiency virus type 1 Tat and methamphetamine affect the release and activation of matrix-degrading proteinases. J Neurovirol 10(1):21–28 14982725 10.1080/13550280490261699

[32] Mahajan SD, Aalinkeel R, Sykes DE, Reynolds JL, Bindukumar B, Fernandez SF, Chawda R, Shanahan TC, Schwartz SA (2008) Tight junction regulation by morphine and HIV-1 tat modulates blood–brain barrier permeability. J Clin Immunol 28:528–541 18574677 10.1007/s10875-008-9208-1

[33] Weiss JM, Nath A, Major EO, Berman JW (1999) HIV-1 Tat induces monocyte chemoattractant protein-1-mediated monocyte transmigration across a model of the human blood-brain barrier and up-regulates CCR5 expression on human monocytes. J Immunol 163(5):2953–2959 10453044

[34] Mishra M, Vetrivel S, Siddappa NB, Ranga U, Seth P (2008) Clade-specific differences in neurotoxicity of human immunodeficiency virus-1 B and C Tat of human neurons: significance of dicysteine C30C31 motif. Annals of neurology 63(3):366–376 18074388 10.1002/ana.21292

[35] Jo H, Jang HY, Youn GS, Kim D, Lee CY, Jang JH, Choi SY, Jun J-G, Park J (2018) Hindsiipropane B alleviates HIV-1 Tat-induced inflammatory responses by suppressing HDAC6-NADPH oxidase-ROS axis in astrocytes. BMB Rep 51(8):394 29699604 10.5483/BMBRep.2018.51.8.061 PMC6130829

[36] Ju SM, Song HY, Lee J, Lee SJ, Choi SY, Park J (2009) Extracellular HIV-1 Tat up-regulates expression of matrix metalloproteinase-9 via a MAPK-NF-κB dependent pathway in human astrocytes. Exp Mol Med 41(2):86–93 19287189 10.3858/emm.2009.41.2.011 PMC2679334

[37] Nath A, Conant K, Chen P, Scott C, Major EO (1999) Transient exposure to HIV-1 Tat protein results in cytokine production in macrophages and astrocytes: a hit and run phenomenon. J Biol Chem 274(24):17098–17102 10358063 10.1074/jbc.274.24.17098

[38] Boven LA, Noorbakhsh F, Bouma G, van der Zee R, Vargas DL, Pardo C, McArthur JC, Nottet HS, Power C (2007) Brain-derived human immunodeficiency virus-1 Tat exerts differential effects on LTR transactivation and neuroimmune activation. J Neurovirol 13(2):173–184 17505986 10.1080/13550280701258399

[39] Nookala AR, Kumar A (2014) Molecular mechanisms involved in HIV-1 Tat-mediated induction of IL-6 and IL-8 in astrocytes. J Neuroinflammation 11:1–18 25539898 10.1186/s12974-014-0214-3 PMC4302610

[40] Nookala AR, Shah A, Noel RJ, Kumar A (2013) HIV-1 Tat-mediated induction of CCL5 in astrocytes involves NF-κB, AP-1, C/EBPα and C/EBPγ transcription factors and JAK, PI3K/Akt and p38 MAPK signaling pathways. PloS one 8(11):e78855 24244375 10.1371/journal.pone.0078855 PMC3823997

[41] Qiu X, Wang J, Zhang W, Duan C, Chen T, Zhang D, Su J, Gao L (2023) Disruption of the ADAM17/NF-κB feedback loop in astrocytes ameliorates HIV-1 Tat-induced inflammatory response and neuronal death. J Neurovirol 29(3):283–296. 10.1007/s13365-023-01131-5 37185939 10.1007/s13365-023-01131-5

[42] Kutsch O, Oh J-W, Nath A, Benveniste E (2000) Induction of the chemokines interleukin-8 and IP-10 by human immunodeficiency virus type 1 tat in astrocytes. J Virol 74(19):9214–9221 10982368 10.1128/jvi.74.19.9214-9221.2000 PMC102120

[43] Mayne M, Bratanich AC, Chen P, Rana F, Nath A, Power C (1998) HIV-1 Tat molecular diversity and induction of TNF-α: implications for HIV-induced neurological disease. Neuroimmunomodulation 5(3–4):184–192 9730685 10.1159/000026336

[44] Williams R, Yao H, Dhillon NK, Buch SJ (2009) HIV-1 Tat co-operates with IFN-γ and TNF-α to increase CXCL10 in human astrocytes. PLoS One 4(5):e5709 19479051 10.1371/journal.pone.0005709 PMC2684622

[45] Likert R (1932) A technique for the measurement of attitudes. Arch Sci Psychol 22(140):55–55

[46] McHugh ML (2012) Interrater reliability: the kappa statistic. Biochem Med (Zagreb) 22(3):276–282 23092060 PMC3900052

[47] Boven LA, Noorbakhsh F, Bouma G, van der Zee R, Vargas DL, Pardo C, McArthur JC, Nottet HS, Power C (2007) Brain-derived human immunodeficiency virus-1 Tat exerts differential effects on LTR transactivation and neuroimmune activation. J Neurovirol 13(2):173–184. 10.1080/13550280701258399 17505986 10.1080/13550280701258399

[48] Kutsch O, Oh J, Nath A, Benveniste EN (2000) Induction of the chemokines interleukin-8 and IP-10 by human immunodeficiency virus type 1 tat in astrocytes. J Virol 74(19):9214–9221. 10.1128/jvi.74.19.9214-9221.2000 10982368 10.1128/jvi.74.19.9214-9221.2000 PMC102120

[49] Mayne M, Bratanich AC, Chen P, Rana F, Nath A, Power C (1998) HIV-1 tat molecular diversity and induction of TNF-alpha: implications for HIV-induced neurological disease. Neuroimmunomodulation 5(3–4):184–192. 10.1159/000026336 9730685 10.1159/000026336

[50] Williams R, Yao H, Dhillon NK, Buch SJ (2009) HIV-1 Tat co-operates with IFN-gamma and TNF-alpha to increase CXCL10 in human astrocytes. PLoS One 4(5):e5709. 10.1371/journal.pone.0005709 19479051 10.1371/journal.pone.0005709 PMC2684622

[51] Jo H, Jang HY, Youn GS, Kim D, Lee CY, Jang JH, Choi SY, Jun JG, Park J (2018) Hindsiipropane B alleviates HIV-1 Tat-induced inflammatory responses by suppressing HDAC6-NADPH oxidase-ROS axis in astrocytes. BMB Rep 51(8):394–399. 10.5483/bmbrep.2018.51.8.061 29699604 10.5483/BMBRep.2018.51.8.061 PMC6130829

[52] Khiati A, Chaloin O, Muller S, Tardieu M, Horellou P (2010) Induction of monocyte chemoattractant protein-1 (MCP-1/CCL2) gene expression by human immunodeficiency virus-1 Tat in human astrocytes is CDK9 dependent. J Neurovirol 16(2):150–167. 10.3109/13550281003735691 20370601 10.3109/13550281003735691

[53] Mishra M, Vetrivel S, Siddappa NB, Ranga U, Seth P (2008) Clade-specific differences in neurotoxicity of human immunodeficiency virus-1 B and C Tat of human neurons: significance of dicysteine C30C31 motif. Ann Neurol 63(3):366–376. 10.1002/ana.21292 18074388 10.1002/ana.21292

[54] Weiss JM, Nath A, Major EO, Berman JW (1999) HIV-1 Tat induces monocyte chemoattractant protein-1-mediated monocyte transmigration across a model of the human blood-brain barrier and up-regulates CCR5 expression on human monocytes. J Immunol 163(5):2953–2959 10453044

[55] Acheampong E, Mukhtar M, Parveen Z, Ngoubilly N, Ahmad N, Patel C, Pomerantz RJ (2002) Ethanol strongly potentiates apoptosis induced by HIV-1 proteins in primary human brain microvascular endothelial cells. Virology 304(2):222–234. 10.1006/viro.2002.1666 12504564 10.1006/viro.2002.1666

[56] Conant K, St Hillaire C, Anderson C, Galey D, Wang J, Nath A (2004) Human immunodeficiency virus type 1 Tat and methamphetamine affect the release and activation of matrix-degrading proteinases. J Neurovirol 10(1):21–28. 10.1080/13550280490261699 14982725 10.1080/13550280490261699

[57] Asia LK, Jansen Van Vuren E, Williams ME (2022) The influence of viral protein R amino acid substitutions on clinical outcomes in people living with HIV: a systematic review. Eur J Clin Invest e13943. 10.1111/eci.13943 10.1111/eci.13943 36579370

[58] Williams ME, Stein DJ, Joska JA, Naudé PJW (2021) Cerebrospinal fluid immune markers and HIV-associated neurocognitive impairments: a systematic review. J Neuroimmunol 358:577649. 10.1016/j.jneuroim.2021.577649 34280844 10.1016/j.jneuroim.2021.577649

[59] Ancuta P, Kamat A, Kunstman KJ, Kim E-Y, Autissier P, Wurcel A, Zaman T, Stone D, Mefford M, Morgello S (2008) Microbial translocation is associated with increased monocyte activation and dementia in AIDS patients. PloS one 3(6):e2516 18575590 10.1371/journal.pone.0002516 PMC2424175

[60] Cohen RA, de la Monte S, Gongvatana A, Ombao H, Gonzalez B, Devlin KN, Navia B, Tashima KT (2011) Plasma cytokine concentrations associated with HIV/hepatitis C coinfection are related to attention, executive and psychomotor functioning. J Neuroimmunol 233(1–2):204–210 21146232 10.1016/j.jneuroim.2010.11.006 PMC3074016

[61] Woods SP, Morgan EE, Marquie-Beck J, Carey CL, Grant I, Letendre SL, Group HNRC (2006) Markers of macrophage activation and axonal injury are associated with prospective memory in HIV-1 disease. Cogn Behav Neurol 19(4):217–221 17159619 10.1097/01.wnn.0000213916.10514.57 PMC1939824

[62] Yuan L, Qiao L, Wei F, Yin J, Liu L, Ji Y, Smith D, Li N, Chen D (2013) Cytokines in CSF correlate with HIV-associated neurocognitive disorders in the post-HAART era in China. J Neurovirol 19:144–149 23389619 10.1007/s13365-013-0150-5 PMC4363104

[63] Xing Y, Shepherd N, Lan J, Li W, Rane S, Gupta SK, Zhang S, Dong J, Yu Q (2017) MMPs/TIMPs imbalances in the peripheral blood and cerebrospinal fluid are associated with the pathogenesis of HIV-1-associated neurocognitive disorders. Brain Behav Immun 65:161–172 28487203 10.1016/j.bbi.2017.04.024 PMC5793222

[64] Eugenin EA, Osiecki K, Lopez L, Goldstein H, Calderon TM, Berman JW (2006) CCL2/monocyte chemoattractant protein-1 mediates enhanced transmigration of human immunodeficiency virus (HIV)-infected leukocytes across the blood–brain barrier: a potential mechanism of HIV–CNS invasion and NeuroAIDS. J Neurosci 26(4):1098–1106 16436595 10.1523/JNEUROSCI.3863-05.2006 PMC6674577

[65] Kim B-H, Hadas E, Kelschenbach J, Chao W, Gu C-J, Potash MJ, Volsky DJ (2023) CCL2 is required for initiation but not persistence of HIV infection mediated neurocognitive disease in mice. Sci Rep 13(1):6577 37085605 10.1038/s41598-023-33491-7 PMC10121554

[66] Williams ME, Ipser JC, Stein DJ, Joska JA, Naudé PJ (2020) Peripheral immune dysregulation in the ART era of HIV-associated neurocognitive impairments: a systematic review. Psychoneuroendocrinology 118:104689 32479968 10.1016/j.psyneuen.2020.104689

[67] Williams ME, Stein DJ, Joska JA, Naudé PJ (2021) Cerebrospinal fluid immune markers and HIV-associated neurocognitive impairments: a systematic review. J Neuroimmunol 358:577649 34280844 10.1016/j.jneuroim.2021.577649

[68] Mishra R, Chhatbar C, Singh SK (2012) HIV-1 Tat C-mediated regulation of tumor necrosis factor receptor-associated factor-3 by microRNA 32 in human microglia. J Neuroinflammation 9(1):131. 10.1186/1742-2094-9-131 22709905 10.1186/1742-2094-9-131 PMC3418571

[69] Nuovo GJ, Alfieri ML, Cerami A (1996) AIDS dementia is associated with massive, activated HIV-1 infection and concomitant expression of several cytokines. Mol Med 2:358–366 8784788 PMC2230156

[70] Fan Y, He JJ (2016) HIV-1 tat promotes lysosomal exocytosis in astrocytes and contributes to astrocyte-mediated tat neurotoxicity. J Biol Chem 291(43):22830–22840 27609518 10.1074/jbc.M116.731836 PMC5077215

[71] Stansley B, Post J, Hensley K (2012) A comparative review of cell culture systems for the study of microglial biology in Alzheimer’s disease. J Neuroinflammation 9(1):115. 10.1186/1742-2094-9-115 22651808 10.1186/1742-2094-9-115 PMC3407712

[72] Du Y, Deng W, Wang Z, Ning M, Zhang W, Zhou Y, Lo EH, Xing C (2017) Differential subnetwork of chemokines/cytokines in human, mouse, and rat brain cells after oxygen–glucose deprivation. J Cereb Blood Flow Metab 37(4):1425–1434 27328691 10.1177/0271678X16656199 PMC5453462

[73] Jung O, Song MJ, Ferrer M (2021) Operationalizing the use of biofabricated tissue models as preclinical screening platforms for drug discovery and development. SLAS Discov 26(9):1164–1176. 10.1177/24725552211030903 34269079 10.1177/24725552211030903 PMC9527638

[74] Mayer T, Jagla B, Wyler MR, Kelly PD, Aulner N, Beard M, Barger G, Többen U, Smith DH, Brandén L, Rothman JE (2006) [15] - Cell‐based assays using primary endothelial cells to study multiple steps in inflammation. In: Inglese J (ed) Methods in enzymology, vol 414. Academic Press, pp 266-283. 10.1016/S0076-6879(06)14015-X 10.1016/S0076-6879(06)14015-X 17110197

[75] Zhou F, Liu X, Gao L, Zhou X, Cao Q, Niu L, Wang J, Zuo D, Li X, Yang Y, Hu M, Yu Y, Tang R, Lee BH, Choi BW, Wang Y, Izumiya Y, Xue M, Zheng K, Gao D (2019) HIV-1 Tat enhances purinergic P2Y4 receptor signaling to mediate inflammatory cytokine production and neuronal damage via PI3K/Akt and ERK MAPK pathways. J Neuroinflammation 16(1):71. 10.1186/s12974-019-1466-8 30947729 10.1186/s12974-019-1466-8 PMC6449963

[76] Youn GS, Kwon D-J, Ju SM, Rhim H, Bae YS, Choi SY, Park J (2014) Celastrol ameliorates HIV-1 Tat-induced inflammatory responses via NF-kappaB and AP-1 inhibition and heme oxygenase-1 induction in astrocytes. Toxicol Appl Pharmacol 280(1):42–52 25064159 10.1016/j.taap.2014.07.010

[77] Bruce-Keller AJ, Barger SW, Moss NI, Pham JT, Keller JN, Nath A (2001) Pro-inflammatory and pro-oxidant properties of the HIV protein Tat in a microglial cell line: attenuation by 17β-estradiol. J Neurochem 78(6):1315–1324 11579140 10.1046/j.1471-4159.2001.00511.x

[78] Santerre M, Wang Y, Arjona S, Allen C, Sawaya BE (2019) Differential contribution of HIV-1 subtypes B and C to neurological disorders: mechanisms and possible treatments. AIDS Rev 21(2):76 31332398 10.24875/AIDSRev.19000051 PMC7219600

[79] Ruiz AP, Ajasin DO, Ramasamy S, DesMarais V, Eugenin EA, Prasad VR (2019) A naturally occurring polymorphism in the HIV-1 tat basic domain inhibits uptake by bystander cells and leads to reduced neuroinflammation. Sci Rep 9(1):3308 30824746 10.1038/s41598-019-39531-5 PMC6397180

[80] Williams ME, Cloete R (2022) Molecular modeling of subtype-specific Tat protein signatures to predict Tat-TAR interactions that may be involved in HIV-associated neurocognitive disorders. Front Microbiol 13:866611 35464972 10.3389/fmicb.2022.866611 PMC9021916

[81] de Almeida SM, Beltrame MP, Tang B, Rotta I, Abramson I, Vaida F, Schrier R, Ellis RJ (2023) Cerebrospinal fluid CD14(++)CD16(+) monocytes in HIV-1 subtype C compared with subtype B. J Neurovirol 29 (3):308–324. 10.1007/s13365-023-01137-z 10.1007/s13365-023-01137-z PMC10769008 37219809

[82] Dara J, Dow A, Cromwell E, Sturdevant CB, Mallewa M, Swanstrom R, Van Rie A, Prasad VR (2015) Multivariable analysis to determine if HIV-1 Tat dicysteine motif is associated with neurodevelopmental delay in HIV-infected children in Malawi. Behav Brain Funct 11:1–8 26678821 10.1186/s12993-015-0083-7 PMC4683967

[83] Mele AR, Marino J, Dampier W, Wigdahl B, Nonnemacher MR (2020) HIV-1 Tat length: comparative and functional considerations. Front Microbiol 11:444. 10.3389/fmicb.2020.00444 32265877 10.3389/fmicb.2020.00444 PMC7105873

