# Supplementary material for: Transactivator of Transcription (Tat)-Induced Neuroinflammation as a Key Pathway in Neuronal Dysfunction: A Scoping Review
Source: Mol Neurobiol. 2024 Apr 17;61(11):9320–46. doi: 10.1007/s12035-024-04173-w (PMC11496333; doi:10.1007/s12035-024-04173-w)
Supplement: Supplementary file 1 — Supplementary file1 (DOCX 13 KB) [file 12035_2024_4173_MOESM1_ESM.docx]

**Pubmed: 316 (30/10/2023):**

(Tat [tw] OR Gene Products, tat [mh] tat Gene Products, Human Immunodeficiency Virus [mh]) AND (microglia [mh] or monocytes [mh] or macrophages [mh] OR astrocytes [mh]) AND (HIV associated neurocognitive disorders [mh] OR HAND [tw] OR neurocognitive [tw] OR cogniti* [tw] OR Neuropsychological Tests [mh] OR neuronal damage [tw] OR neuronal apoptosis [tw] OR inflammation [mh] OR Cytokines [mh] OR Chemokines [mh] OR Neurogenic Inflammation [mh] OR neuroinflammation [tw] OR TNF [tw] OR Interleukins [mh] OR interleukins [tw] OR Microglia [mh] OR Monocytes [mh] OR Microglia [mh] OR microglia [tw] OR Monocytes [mh] OR monocyte* [tw] OR sCD163 [tw] OR sCD14 [tw] OR sCD40 [tw] OR Neopterin [mh] OR Interferons [mh])

**Web of science: 442 (30/10/2023):**

TS=(Transactivation of transcription OR Tat or Tat protein or Tat peptide) AND TS=(HIV associated neurocognitive disorders OR HAND OR neurocognitive OR cogniti* OR Neuropsychological Tests OR neuronal damage OR neuronal apoptosis) AND TS=(Cytokines OR cytokin* OR Chemokines OR chemokine OR Inflammation OR inflammation OR Neurogenic Inflammation OR neuroinflammation OR TNF OR Interleukins OR interleukins OR Microglia OR microglia OR Monocytes OR monocyte* OR sCD163 OR sCD14 OR sCD40 OR neopterin OR interferons)

**Scopus: 267 (30/10/2023):**

(Tat or transactivation of transcription or Tat protein or Tat peptide) AND (hiv associated neurocognitive disorders OR hand OR neurocognitive OR cogniti* OR neuropsychological tests OR neuronal damage OR neuronal apoptosis) AND (cytokines OR cytokin* OR chemokines OR chemokine OR inflammation OR neurogenic inflammation OR neuroinflammation OR tnf OR interleukins OR interleukin OR microglia OR monocytes OR monocyte*OR scd163 OR scd14 OR scd40 OR neopterin OR interferons)

**Total 1025**
