# Supplementary material for: Transactivator of Transcription (Tat)-Induced Neuroinflammation as a Key Pathway in Neuronal Dysfunction: A Scoping Review
Source: Mol Neurobiol. 2024 Apr 17;61(11):9320–46. doi: 10.1007/s12035-024-04173-w (PMC11496333; doi:10.1007/s12035-024-04173-w)
Supplement: Supplementary file 2 — Supplementary file2 (DOCX 43 KB) [file 12035_2024_4173_MOESM2_ESM.docx]

**Supplementary table 1**: Quality control of included studies conducted by TM.

| **Reference** | **Question 1** | **Question 2** | **Question 3** | **Question 4** | **Rating** |
| --- | --- | --- | --- | --- | --- |
| ^(Acheampong et al., 2002)^ | 2 | 2 | 2 | 2 | High |
| ^(Boven et al., 2007)^ | 2 | 2 | 2 | 2 | High |
| ^(Conant et al., 1998)^ | 2 | 2 | 1 | 1 | High |
| ^(Conant et al., 2004)^ | 2 | 2 | 1 | 1 | High |
| ^(Aversa et al., 2004)^ | 2 | 2 | 2 | 2 | High |
| ^(Gao et al., 2023)^ | 2 | 2 | 2 | 2 | High |
| ^(Jo et al., 2018)^ | 2 | 2 | 2 | 2 | High |
| ^(Ju et al., 2009)^ | 2 | 2 | 2 | 2 | High |
| ^(Khiati et al., 2010)^ | 2 | 2 | 2 | 2 | High |
| ^(Kutsch et al., 2000)^ | 2 | 2 | 2 | 2 | High |
| ^(Mahajan et al., 2008)^ | 2 | 2 | 1 | 2 | High |
| ^(Mayne et al., 1998)^ | 2 | 2 | 1 | 2 | High |
| ^(Mishra et al., 2008)^ | 2 | 2 | 2 | 2 | High |
| ^(Nath et al., 1999)^ | 2 | 2 | 2 | 2 | High |
| ^(Nookala and Kumar, 2014)^ | 2 | 2 | 2 | 2 | High |
| ^(Nookala et al., 2013)^ | 2 | 2 | 2 | 2 | High |
| ^(Qiu et al., 2023)^ | 2 | 2 | 2 | 2 | High |
| ^(Sheng et al., 2000)^ | 2 | 2 | 2 | 2 | High |
| ^(Sui et al., 2007)^ | 2 | 2 | 2 | 2 | High |
| ^(Tewari et al., 2015)^ | 2 | 2 | 1 | 2 | High |
| ^(Weiss et al., 1999)^ | 2 | 2 | 2 | 2 | High |
| ^(Williams et al., 2009)^ | 2 | 2 | 1 | 2 | High |

**Supplementary table 1**: Quality control of included studies conducted by MW

| **Reference** | **Question 1** | **Question 2** | **Question 3** | **Question 4** | **Rating** |
| --- | --- | --- | --- | --- | --- |
| ^(Acheampong et al., 2002)^ | 2 | 2 | 2 | 2 | High |
| ^(Boven et al., 2007)^ | 1 | 2 | 1 | 2 | High |
| ^(Conant et al., 1998)^ | 1 | 2 | 2 | 2 | High |
| ^(Conant et al., 2004)^ | 2 | 2 | 1 | 1 | High |
| ^(Aversa et al., 2004)^ | 2 | 2 | 2 | 2 | High |
| ^(Gao et al., 2023)^ | 2 | 2 | 2 | 2 | High |
| ^(Jo et al., 2018)^ | 1 | 2 | 2 | 2 | High |
| ^(Ju et al., 2009)^ | 2 | 2 | 2 | 2 | High |
| ^(Khiati et al., 2010)^ | 2 | 2 | 2 | 2 | High |
| ^(Kutsch et al., 2000)^ | 2 | 2 | 2 | 2 | High |
| ^(Mahajan et al., 2008)^ | 1 | 2 | 1 | 2 | High |
| ^(Mayne et al., 1998)^ | 1 | 2 | 1 | 2 | High |
| ^(Mishra et al., 2008)^ | 2 | 2 | 2 | 2 | High |
| ^(Nath et al., 1999)^ | 2 | 2 | 2 | 2 | High |
| ^(Nookala and Kumar, 2014)^ | 2 | 2 | 2 | 2 | High |
| ^(Nookala et al., 2013)^ | 2 | 2 | 2 | 2 | High |
| ^(Qiu et al., 2023)^ | 2 | 2 | 2 | 2 | High |
| ^(Sheng et al., 2000)^ | 2 | 2 | 2 | 2 | High |
| ^(Sui et al., 2007)^ | 1 | 2 | 1 | 2 | High |
| ^(Tewari et al., 2015)^ | 2 | 2 | 1 | 2 | High |
| ^(Weiss et al., 1999)^ | 1 | 2 | 2 | 2 | High |
| ^(Williams et al., 2009)^ | 1 | 2 | 1 | 2 | High |

Questions 1-5 were classified as follows: 1) Is it clear in the study what is the ‘cause’ and what is the ‘effect’ (i.e., there is no confusion about which variable comes first)? 2) Was there a control group? 3) Were there multiple measurements of the outcome both pre and post the intervention/exposure? And 4) Were outcomes measured in a reliable way? Studies with ratings between 6 and 8 were classified as high quality. Studies with ratings between 3 and 5 were considered as intermediate quality, and between 0 and 2 as low quality.
